# Supplementary material for: CK2 phosphorylation of CMTR1 promotes RNA cap formation and influenza virus infection
Source: Cell Rep. 2024 Jun 25;43(7):114405. doi: 10.1016/j.celrep.2024.114405 (PMC11290353; doi:10.1016/j.celrep.2024.114405)
Supplement: Document S1. Figures S1–S6 [file mmc1.pdf]

Cell Reports, Volume *43*

Supplemental information

CK2 phosphorylation of CMTR1 promotes RNA cap formation and influenza virus infection

Radoslaw Lukoszek, Francisco Inesta-Vaquera, Natasha J.M. Brett, Shang Liang, Lydia A. Hepburn, David J. Hughes, Chiara Pirillo, Edward W. Roberts, and Victoria H. Cowling

A

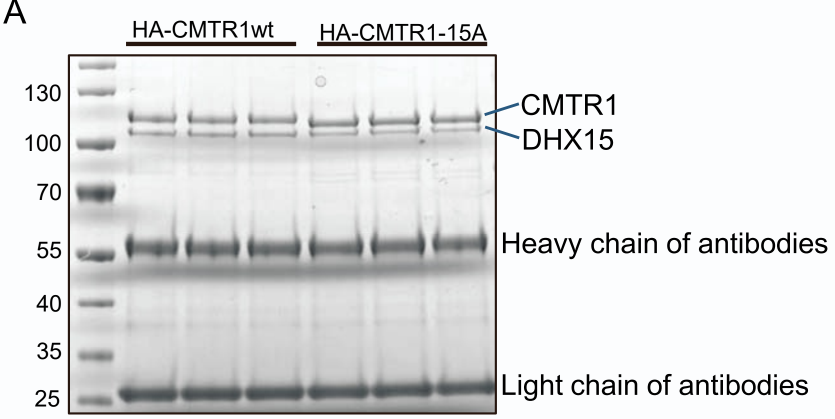

B

HA-CMTR1 WT peptide coverage (90% coverage)

1	MYPYDVPDYA	MKRRTDPECT	APIKKQKKRV	AELALSLSST	SDDEPPSSVS
51	HGAKASTTSL	SGSDSETEGK	QHSSDSFDDA	FKADSLVEGT	SSRYSMYNSV
101	SQKLMAKMGF	REGEGLGKYS	QGRKDIVEAS	SQKGRRGLGL	TLRGFDQELN
151	VDWRDEPEPS	ACEQVSWFPE	CTTEIPDTQE	MSDWMVVGKR	KMIIEDETEF
201	CGEELLHSVL	QCKSVFDVLD	GEEMRRARTR	ANPYEMIRGV	FFLNRAAMKM
251	ANMDFVFDRM	FTNPRDSYGK	PLVKDREAEL	LYFADVCAGP	GGFSEYVLWR
301	KKWHAKGFGM	TLKGPNDFKL	EDFYSASSEL	FEPYYGEGGI	DGDGDITRPE
351	NISAFRNFVL	DNTDRKGVHF	LMADGGFSVE	GQENLQEILS	KQLLLCQFLM
401	ALSIVRTGGH	FICKTFDLFT	PFSVGLVYLL	YCCFERVCLF	KPITSRPANS
451	ERYVVCKGLK	VGIDDVRDYL	FAVNIKLNQL	RNTDSDVNLV	VPLEVIKGDH
501	EFTDYMIRSN	ESHCSLQIKA	LAKIHAFVQD	TTLSEPRQAE	IRKECLRLWG
551	IPDQARVAPS	SSDPKSKFFE	LIQGTEIDIF	SYKPTLLTSK	TLEKIRPVFD
601	YRCMVSGSEQ	KFLIGLGKSQ	IYTWDGRQSD	RWIKLDLKTE	LPRDTLLSVE
651	IVHELKGEGK	AQRKISAIHI	LDVLVLNGTD	VREQHFNQRI	QLAEKFVKAV
701	SKPSRPDMNP	IRVKEVYRLE	EMEKIFVRLE	MKIIKGSSGT	PKLSYTGRDD
751	RHFVPMGLYI	VRTVNEPWTM	GFSKSFKKKF	FYNKKTKDST	FDLPADSIAP
801	FHICYYGRLF	WEWGDGIRVH	DSQKPQDQDK	LSKEDVLSFI	QMHRA

HA-CMTR1 15A peptide coverage (91% coverage)

1	MYPYDVPDYA	MKRRTDPECT	APIKKQKKRV	AELALALASA	ADDEPPSSVS
51	HGAKAATTAL	AGADAEAEGK	QHAADAFDDA	FKADALVEGT	SSRYSMYNAV
101	SQKLMAKMGF	REGEGLGKYS	QGRKDIVEAS	SQKGRRGLGL	TLRGFDQELN
151	VDWRDEPEPS	ACEQVSWFPE	CTTEIPDTQE	MSDWMVVGKR	KMIIEDETEF
201	CGEELLHSVL	QCKSVFDVLD	GEEMRRARTR	ANPYEMIRGV	FFLNRAAMKM
251	ANMDFVFDRM	FTNPRDSYGK	PLVKDREAEL	LYFADVCAGP	GGFSEYVLWR
301	KKWHAKGFGM	TLKGPNDFKL	EDFYSASSEL	FEPYYGEGGI	DGDGDITRPE
351	NISAFRNFVL	DNTDRKGVHF	LMADGGFSVE	GQENLQEILS	KQLLLCQFLM
401	ALSIVRTGGH	FICKTFDLFT	PFSVGLVYLL	YCCFERVCLF	KPITSRPANS
451	ERYVVCKGLK	VGIDDVRDYL	FAVNIKLNQL	RNTDSDVNLV	VPLEVIKGDH
501	EFTDYMIRSN	ESHCSLQIKA	LAKIHAFVQD	TTLSEPRQAE	IRKECLRLWG
551	IPDQARVAPS	SSDPKSKFFE	LIQGTEIDIF	SYKPTLLTSK	TLEKIRPVFD
601	YRCMVSGSEQ	KFLIGLGKSQ	IYTWDGRQSD	RWIKLDLKTE	LPRDTLLSVE
651	IVHELKGEGK	AQRKISAIHI	LDVLVLNGTD	VREQHFNQRI	QLAEKFVKAV
701	SKPSRPDMNP	IRVKEVYRLE	EMEKIFVRLE	MKIIKGSSGT	PKLSYTGRDD
751	RHFVPMGLYI	VRTVNEPWTM	GFSKSFKKKF	FYNKKTKDST	FDLPADSIAP
801	FHICYYGRLF	WEWGDGIRVH	DSQKPQDQDK	LSKEDVLSFI	QMHRA

Figure S1, related to Figure 1. Peptide coverage in CMTR1 mass spectrometry analysis

Human HA-CMTR1 WT and 15A (mutant with 15 alanine substitutions, see text) were expressed in HeLa cells, immunoprecipitated via the HA tag and analysed by mass spectrometry. A) SDS PAGE analysis. Protein visualized by Coomassie stain. CMTR1 and binding partner DHX15 indicated. For each (IP), 3 replicates were performed. B) The peptides identified are indicated in red on the human CMTR1 amino acid sequence.

HA-CMTR1 T30A, S31A, S51A, S53A, S55A, T57A, S66A				
**Peptides Identified by mass spectrometry**	**MH+ [Da]**	**Mascot score**	**Possible sites**	**% Probability**
YSMYNSVSQK	1286.50957	48	S89	98.9
ASTTSLAGADAEAEGK	1558.65837	53	S51	84.2
VAELALSLSSAADDEPPSSVSHGAK	2518.16196	85	S26	99.9
QHSSDAFDDAFKADSLVEGTSSR	2550.06882	28	S75	100

HA-CMTR1 S26A, S28A, T30A, S31A, S51A, S53A, S55A, T57A, S64A, S66A, S75A, S89A				
**Peptides Identified by mass spectrometry**	**MH+ [Da]**	**Mascot score**	**Possible sites**	**% Probability**
ASTTSLAGADAEAEGK	1558.66814	49	S49	99.3
ASTTSLAGADAEAEGKQHSADAFDDAFK	2891.25034	34	S63	100

HA-CMTR1 S26A, S28A, T30A, S31A, S46A, S49A, S51A, S53A, S55A, T57A, S63A, S64A, S66A, S75A, S89A (15A)				
**Peptides Identified by mass spectrometry**	**MH+ [Da]**	**Mascot score**	**Possible sites**	**% Probability**
No phosphorylated peptides detected.				

Figure S2, related to Figure 1. CMTR1 phospho-site mapping

The HA-CMTR1 alanine mutants indicated were transiently expressed in HeLa cells and immunoprecipitated via the HA tag. Phopho-peptides were identified by mass spectrometry, mass reported. The most likely phosphorylation sites are stated in “possible sites” with % probability given.

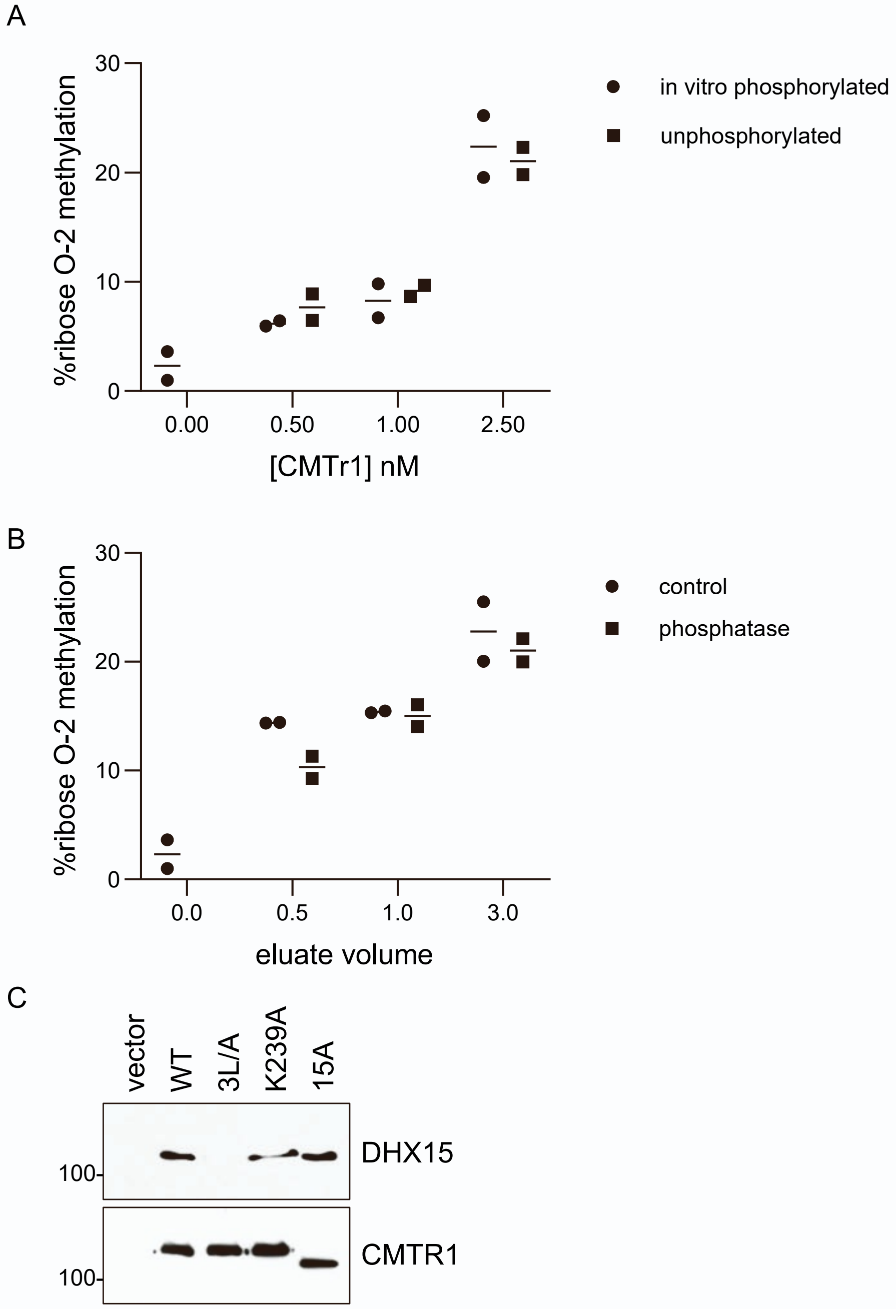

Figure S3, related to Figure 2. CMTR1 phosphorylation does not significantly alter methyltransferase activity or DHX15 interaction

A) Recombinant CMTR1 was phosphorylated *in vitro* by CK2 or mock control (see methods). B) HA-CMTR1 was immunoprecipitated from HeLa cells, eluted and subject to Lambda phosphatase treatment or mock control. For A and B) Ribose O-2 methylation was quantified by *in vitro* assay. Bar represents the average. C) HA-CMTR1 WT, 3L/A, K239A (catalytic dead mutant) and 15A were transiently expressed in HeLa cells. HA- CMTR1 complexes were immunoprecipitated and analysed by western blot for CMTR1 and DHX15 components.

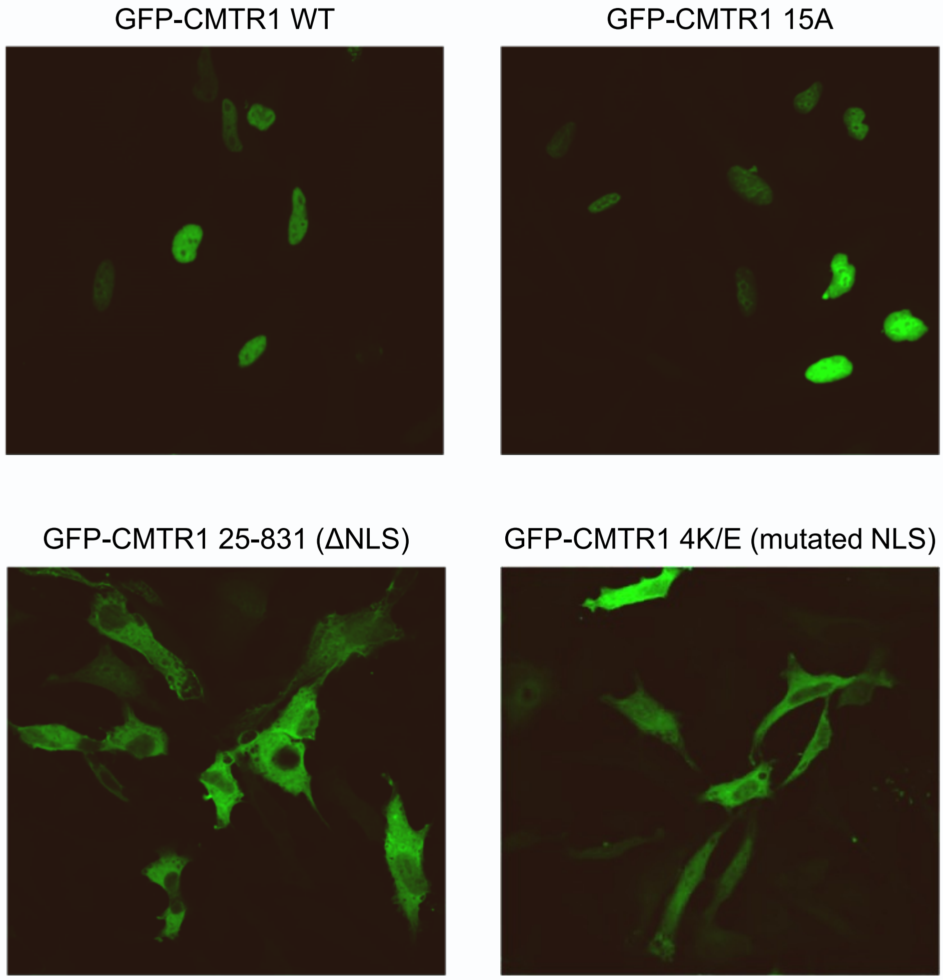

Figure S4, related to Figure 2. GFP-CMTR1 WT and 15A have a diffuse nuclear localisation. GFP-CMTR1 WT, 15A, 25-831 (ΔNLS, nuclear localisation sequence) and 4K/E (mutated NLS) were expressed in HeLa cells and visualised by fluorescence microscopy.

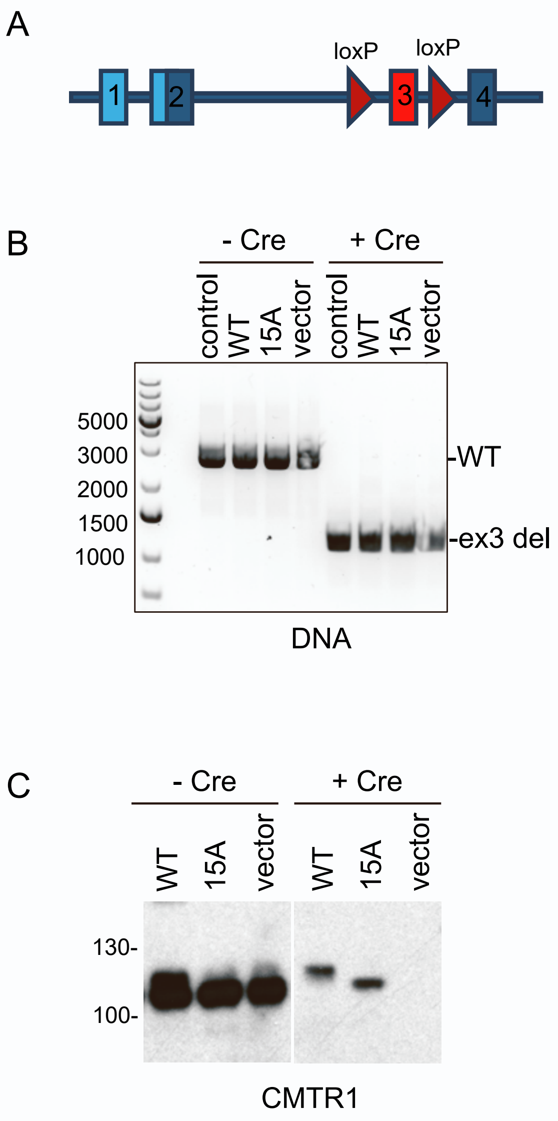

Figure S5, related to Figure 5. Cmtr1 floxed gene

a) Diagram of floxed *Cmtr1* gene in mouse embryonic fibroblasts (MEFs). Exons numbered and loxP sites indicated. Exon 3 (red) is deleted by expression of Cre recombinase, resulting in a frameshift. MEF lines were created to express CMTR1 WT, 15A and vector control. Cre recombinase was expressed transiently. b) PCR of genomic DNA surrounding exon 3. WT product and exon 3 deleted product after expression of Cre recombinases indicated. c) Cell extracts were analysed for CMTR1 expression by western blot before (-Cre) and after (+Cre) Cre mediated deletion. The CMTR1 antibody recognises endogenous CMTR1 and HA-CMTR1.

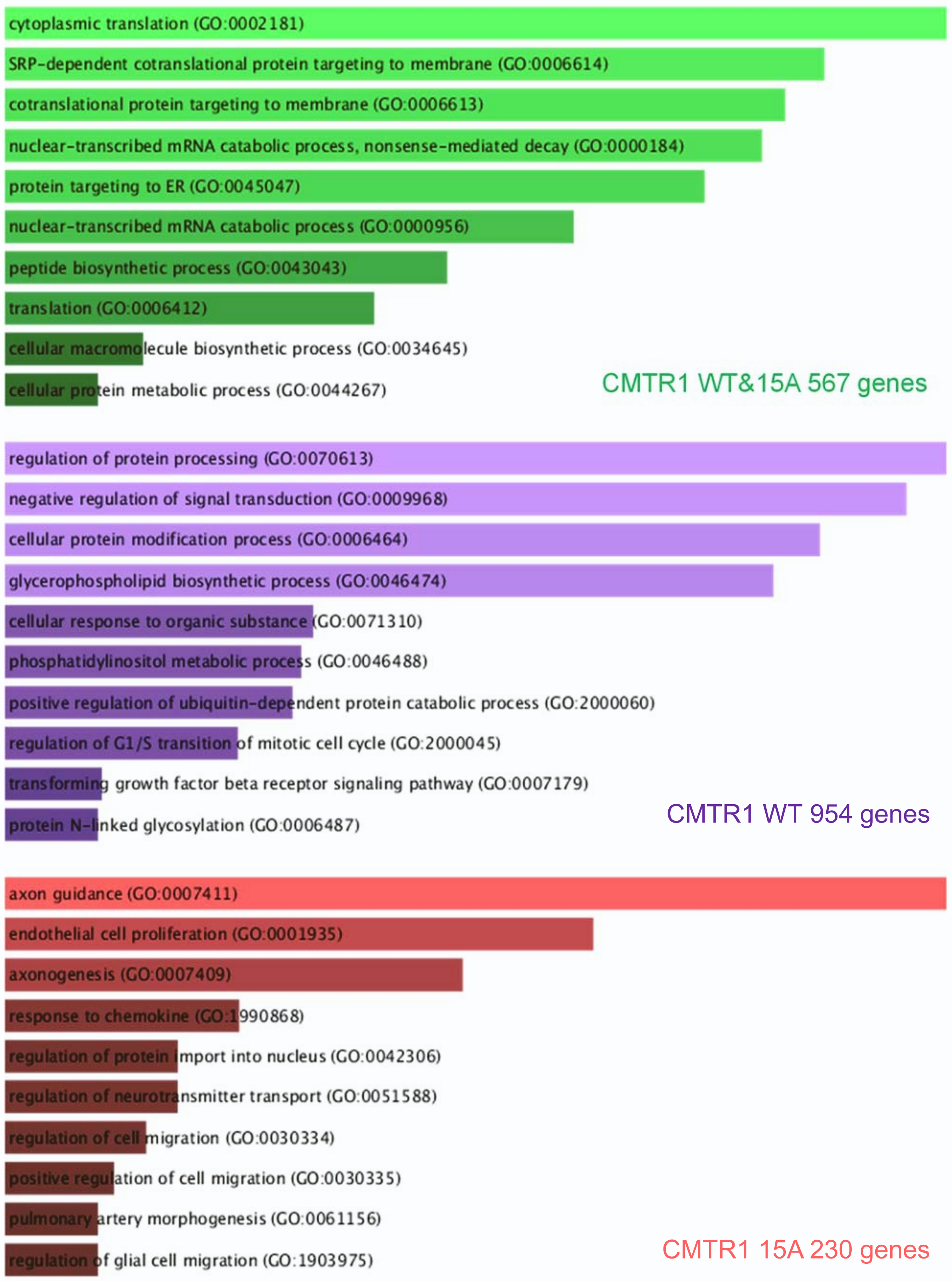

Figure S6, related to Figure 5. GO Term analysis of CMTR1 regulated genes

Genes regulated in response to CMTR1 WT, CMTR1 15A or both in Cmtr1 -/- MEFs. The number of genes in each data set indicated.
