# Supplementary material for: CK2 phosphorylation of CMTR1 promotes RNA cap formation and influenza virus infection
Source: Cell Rep. 2024 Jun 25;43(7):114405. doi: 10.1016/j.celrep.2024.114405 (PMC11290353; doi:10.1016/j.celrep.2024.114405)
Supplement: Document S2. Article plus supplemental information [file mmc3.pdf]

Cell Reports

Article

CK2 phosphorylation of CMTR1 promotes RNA cap formation and influenza virus infection

Graphical abstract

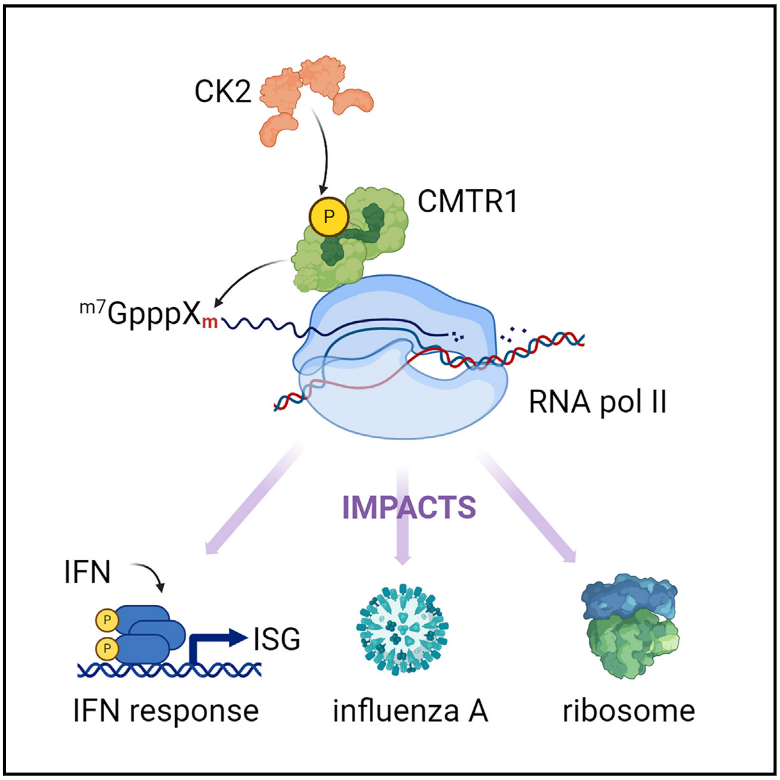

Authors

Radoslaw Lukoszek, Francisco Inesta-Vaquera, Natasha J.M. Brett, ..., Chiara Pirillo, Edward W. Roberts, Victoria H. Cowling

Correspondence

victoria.cowling@glasgow.ac.uk

In brief

Lukozek et al. report that the RNA cap methyltransferase CMTR1 is phosphorylated by CK2, which promotes the interaction with RNA Pol II and RNA cap formation. CMTR1 phosphorylation is required for ribosomal protein gene expression and the interferon response. Propagation of the cap-snatching influenza virus depends on host CMTR1 phosphorylation.

Highlights

- CK2 phosphorylates the RNA cap methyltransferase CMTR1, resulting in binding to RNA Pol II
- CMTR1 P-Patch phosphorylation is required for RNA cap methylation and gene expression
- Expression of interferon-stimulated genes (ISGs) is dependent on CMTR1 P-Patch phosphorylation
- Influenza A, a cap-snatching virus, is dependent on CMTR1 phosphorylation for infection

Lukoszek et al., 2024, Cell Reports *43*, 114405
July 23, 2024 © 2024 The Author(s). Published by Elsevier Inc.
https://doi.org/10.1016/j.celrep.2024.114405

Cell Reports

CellPress
OPEN ACCESS

Article

CK2 phosphorylation of CMTR1 promotes RNA cap formation and influenza virus infection

Radoslaw Lukoszek,[1,6] Francisco Inesta-Vaquera,[1,2,6] Natasha J.M. Brett,[1,3,4,6] Shang Liang,[3] Lydia A. Hepburn,[3] David J. Hughes,[5] Chiara Pirillo,[3] Edward W. Roberts,[3,4] and Victoria H. Cowling[1,3,4,7,*]
[1]School of Life Sciences, University of Dundee, Dundee DD1 5EH, UK
[2]Department of Biochemistry and Molecular Biology and Genetics, School of Sciences, Universidad de Extremadura, Avenida de Elvas, s/n, 06006 Badajoz, Spain
[3]Cancer Research UK Scotland Institute, Garscube Estate, Switchback Road, Glasgow G61 1BD, UK
[4]School of Cancer Sciences, University of Glasgow, Garscube Estate, Switchback Road, Glasgow G61 1QH, UK
[5]School of Biology, University of St Andrews, Biomedical Sciences Research Complex, St Andrews KY16 9ST, UK
[6]These authors contributed equally
[7]Lead contact
*Correspondence: victoria.cowling@glasgow.ac.uk
https://doi.org/10.1016/j.celrep.2024.114405

SUMMARY

The RNA cap methyltransferase CMTR1 methylates the first transcribed nucleotide of RNA polymerase II transcripts, impacting gene expression mechanisms, including during innate immune responses. Using mass spectrometry, we identify a multiply phosphorylated region of CMTR1 (phospho-patch [P-Patch]), which is a substrate for the kinase CK2 (casein kinase II). CMTR1 phosphorylation alters intramolecular interactions, increases recruitment to RNA polymerase II, and promotes RNA cap methylation. P-Patch phosphorylation occurs during the G1 phase of the cell cycle, recruiting CMTR1 to RNA polymerase II during a period of rapid transcription and RNA cap formation. CMTR1 phosphorylation is required for the expression of specific RNAs, including ribosomal protein gene transcripts, and promotes cell proliferation. CMTR1 phosphorylation is also required for interferon-stimulated gene expression. The cap-snatching virus, influenza A, utilizes host CMTR1 phosphorylation to produce the caps required for virus production and infection. We present an RNA cap methylation control mechanism whereby CK2 controls CMTR1, enhancing co-transcriptional capping.

INTRODUCTION

CMTR1 (cap methyltransferase 1) is an RNA cap methyltransferase that has influential roles in gene expression and innate immune responses.[1,2] During pre-mRNA maturation, CMTR1 methylates the first transcribed nucleotide ribose at the O-2 position, creating the RNA cap modification N1 2′-*O*-Me, which alters the affinity of the RNA cap for interacting proteins.[3–6] An absence of cap N1 2′-*O*-Me contributes to mRNA being detected as “non-self”; these immature caps interact with proteins that target RNA for decapping and degradation.[2,5,7] Once the RNA cap has N1 2′-*O*-Me, interactions with proteins of the RNA degradation pathway decrease, and interactions with proteins involved in RNA processing and translation factors alter, associated with the increased expression of specific genes.[2,8]

CMTR1 was first investigated as an interferon-stimulated gene (ISG95)[9–13]; the interferon-induced translation of select ISGs was found to be dependent on this RNA cap methyltransferase.[13] CMTR1 is also upregulated during embryonic stem cell differentiation and is critical for the proliferation of differentiating cells in a mechanism linked to the expression of histone and ribosomal protein gene transcripts.[14,15] Across diverse species, specific genes are responsive to CMTR1 levels, with regulation observed at the level of RNA and translation.[1,8,14,16–22]

The methyltransferase domain is centrally positioned in CMTR1, flanked by domains that influence the interactions and activity of the enzyme. A nuclear localization signal and the G-Patch (glycine-rich) domain are N-terminal to the methyltransferase domain with the non-catalytic guanylyltransferase-like (GT-like) and WW domains residing at the C terminus[3,4] (Figure 1). CMTR1 methylates RNA caps during transcription when the CMTR1 WW domain interacts with the RNA polymerase II (RNA Pol II) large subunit C-terminal domain (CTD) phosphorylated on serine-5 (S5P)[14,18,23] and the CMTR1 GT-like domain interacts with RNA Pol II RPB7.[24] These interactions recruit CMTR1 to RNA Pol II at the initiation of transcription after guanosine cap addition.[14,24] Interaction with RNA may also aid the recruitment of CMTR1 to RNA Pol II.[20] Repression of CMTR1 results in a loss of RNA Pol II binding to the transcription start site and repression of transcription, indicating a role for this enzyme

This is an open access article under the CC BY license (http://creativecommons.org/licenses/by/4.0/).

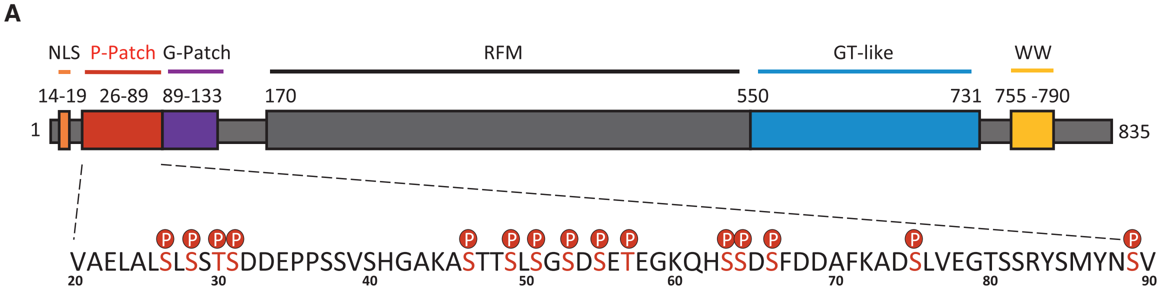

B

HA-CMTR1				
**Peptides Identified by mass spectrometry**	MH+ [Da]	Mascot score	Possible sites	% Probability
VAELALSLSSTSDDEPPSSVSHGAK	2564.16996	51	T30	90.2
QHSSDSFDDAFKADSLVEGTSSR	2566.06620	24	S66	98.1
VAELALSLSSTSDDEPPSSVSHGAK	2644.13431	52	S28 / S29	91.2, 36.2
RVAELALSLSSTSDDEPPSSVSHGAK	2720.26792	53	S31	98.9
RVAELALSLSSTSDDEPPSSVSHGAK	2800.23648	38	S28 or S29	94.2, 78.5
ASTTSLSGSDSETEGKQHSSDSFDDAFK	3081.17759	29	S51, S53	multi
ASTTSLSGSDSETEGKQHSSDSFDDAFKADSLVEG TSSR	3161.14450	25	S51 / S53 / S55	multi

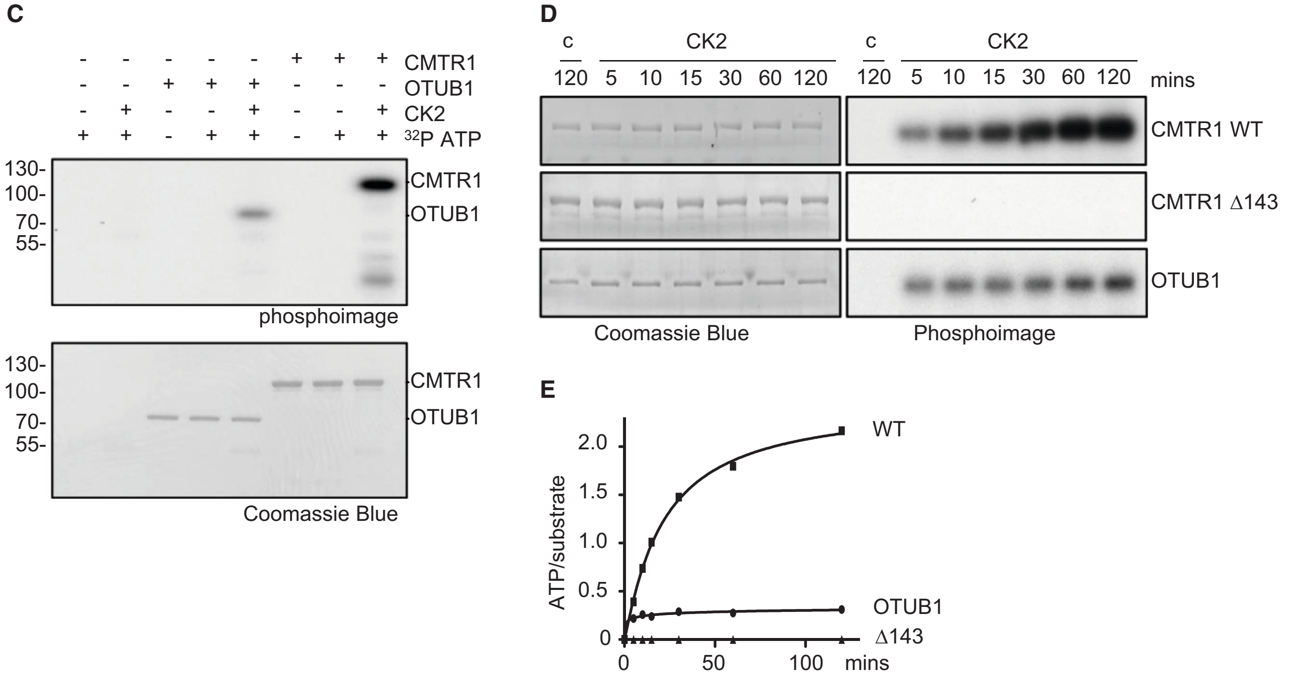

(legend on next page)

and the RNA cap modification, N1 2′-*O*-Me, in transcription and/or co-transcriptional RNA stability.[14,15] The genes with the highest CMTR1 and RNA Pol II binding are the most responsive to the repression of CMTR1. The CMTR1 G-Patch domain also binds directly to the DHX15 helicase (DEAH-box helicase 15) through the OB-fold (oligonucleotide/oligosaccharide-binding), an interaction that modulates CMTR1 and DHX15 activities and prevents interaction with RNA Pol II.[18,19,22]

Here, we demonstrate that CMTR1 function is controlled by the kinase CK2 (casein kinase II). CK2 phosphorylates CMTR1 on multiple residues in the N-terminal phospho-patch (P-Patch), which alters intramolecular interactions and promotes recruitment to the RNA Pol II CTD. CMTR1 P-Patch phosphorylation is required for mature cap formation, specific gene expression, and cell proliferation. The expression of ribosomal protein genes and select ISGs is particularly dependent on CMTR1 phosphorylation. The cap-snatching virus, influenza A virus (IAV), requires host cell CMTR1 phosphorylation for viral infection.

RESULTS

CK2 phosphorylates CMTR1 on multiple amino acids in the N-terminal P-Patch

To identify signaling pathways that influence RNA cap formation, we analyzed phosphorylated residues of the cap methyltransferase CMTR1. Human hemagglutinin (HA)-CMTR1 was expressed in HeLa cells, immunoprecipitated, and analyzed by LC-MS (liquid chromatography-mass spectrometry) (Figures 1A, 1B, and S1A). A cluster of phosphorylated amino acids was identified in an N-terminal region, which we named the P-Patch (amino acids S26–S89; Figures 1A, 1B, and S1B). The phosphorylated amino acids identified with the highest confidence were S28, T30, S31, and S66 (Figures 1A and 1B). The *CMTR1* cDNA was mutated to encode alanine in substitution of phosphorylated amino acids, and, as previously, this CMTR1 protein was expressed in cells and analyzed by LC-MS, resulting in the identification of additional phosphorylation sites (Figures 1A and S2). This process was repeated until 15 phosphorylated amino acids were identified: S26, S28, T30, S31, S46, S49, S51, S53, S55, T57, S63, S64, S66, S75, and S89 (Figures 1A, S1, and S2). HA-CMTR1 15A cDNA was generated to encode CMTR1 with all detected phosphorylated amino acids mutated to alanine. No phosphorylation was detected in HA-CMTR1 15A, despite 91% protein being analyzed by LC-MS, including the entire P-Patch (Figures S1B and S2).

We investigated the kinases that phosphorylate the CMTR1 P-Patch based on consensus motifs. CK2, a pleiotropic kinase that phosphorylates serines and threonines upstream of acidic residues, was a candidate P-Patch kinase.[25–28] Recombinant CMTR1 was phosphorylated by recombinant CK2 *in vitro* (Figure 1C). OTUB1, an established CK2 substrate, was included as a positive control.[29] CMTR1Δ1–143 (lacking the nuclear localization sequence [NLS], P-Patch, and G-Patch) was not phosphorylated in this assay, consistent with the CK2 phosphorylation sites being restricted to the N terminus of CMTR1 (Figures 1D and 1E). More than 2 mol of phosphate was incorporated into each mole of CMTR1, indicating that CMTR1 proteins were multiply phosphorylated (Figure 1E).

To characterize CMTR1 phosphorylation, polyclonal antibodies were raised against a CMTR1 peptide phosphorylated on S28, T30, and S31. In a dot blot, the anti-pCMTR1 (phospho-CMTR1) antibody had enhanced affinity for recombinant CMTR1 when phosphorylated by CK2 (Figure 2A). When HA-CMTR1 was immunoprecipitated from cell extracts, the pCMTR1 antibody bound with higher affinity to the wild-type (WT) protein compared to the 15A mutant, consistent with WT CMTR1 phosphorylation (Figure 2B). Transfection of HeLa cells with a CK2 expression vector resulted in increased endogenous pCMTR1, and transfection with kinase-dead CK2 (CK2-KD) resulted in reduced pCMTR1, consistent with CK2 phosphorylation of CMTR1 in cells (Figure 2C).

CMTR1 is phosphorylated predominantly during G1 phase

The guanosine cap is methylated by RNMT predominantly during G1 phase of the cell cycle.[30] We used nocodazole-based cell synchronization, which releases cells from a late G2/M arrest into G1 phase to determine that CMTR1 phosphorylation increases during G1 phase of the cell cycle (Figures 2D–2F). CMTR1 phosphorylation (ratio of pCMTR1 to CMTR1) peaked at 2 h following the release from nocodazole block, whereas total CMTR1 levels were not altered. Once the phase of the cell cycle when CMTR1 is phosphorylated was determined, we could further verify the pCMTR1 antibody as phosphate specific. CMTR1 was immunoprecipitated from cells 2 h after nocodazole release, and immunoprecipitates were treated with lambda phosphatase, resulting in reduced pCMTR1 levels (Figure 2G). To verify that CK2 was a kinase responsible for CMTR1 phosphorylation during the cell cycle, cells were treated with quinalizarin, a CK2 inhibitor, which reduced detection of pCMTR1 (Figures 2H and 2I).[31]

CMTR1 phosphorylation increases interaction with RNA Pol II

We investigated the impact of P-Patch phosphorylation on CMTR1 function. *In vitro*, CK2 phosphorylation of recombinant

Figure 1. CMTR1 contains a highly phosphorylated P-Patch at the N terminus

(A) Diagram of human CMTR1. Domains and positions indicated. P-Patch is amino acids 26–89 (red). High-confidence phosphorylation sites are labeled "P."

(B) HA-CMTR1 was expressed in HeLa cells and immunoprecipitated via the HA tag. Phospho-peptides were identified by mass spectrometry, and the mass was reported. High-confidence phosphorylation sites are stated in "possible sites" with the percentage of probability given. For peptides with several potential sites, the most likely are stated and all are underlined.

(C) *In vitro* phosphorylation of recombinant CMTR1 and OTUB1 by CK2. Reaction constituents indicated. Phospho-analysis of proteins (top). Coomassie blue staining of proteins (bottom).

(D) *In vitro* phosphorylation of recombinant CMTR1, CMTR1Δ143, and OTUB1 over a time course, as above. "c" indicates a reaction without CK2.

(E) Quantitation of moles of ATP incorporated into moles of substrates in (D).

See also Figures S1 and S2.

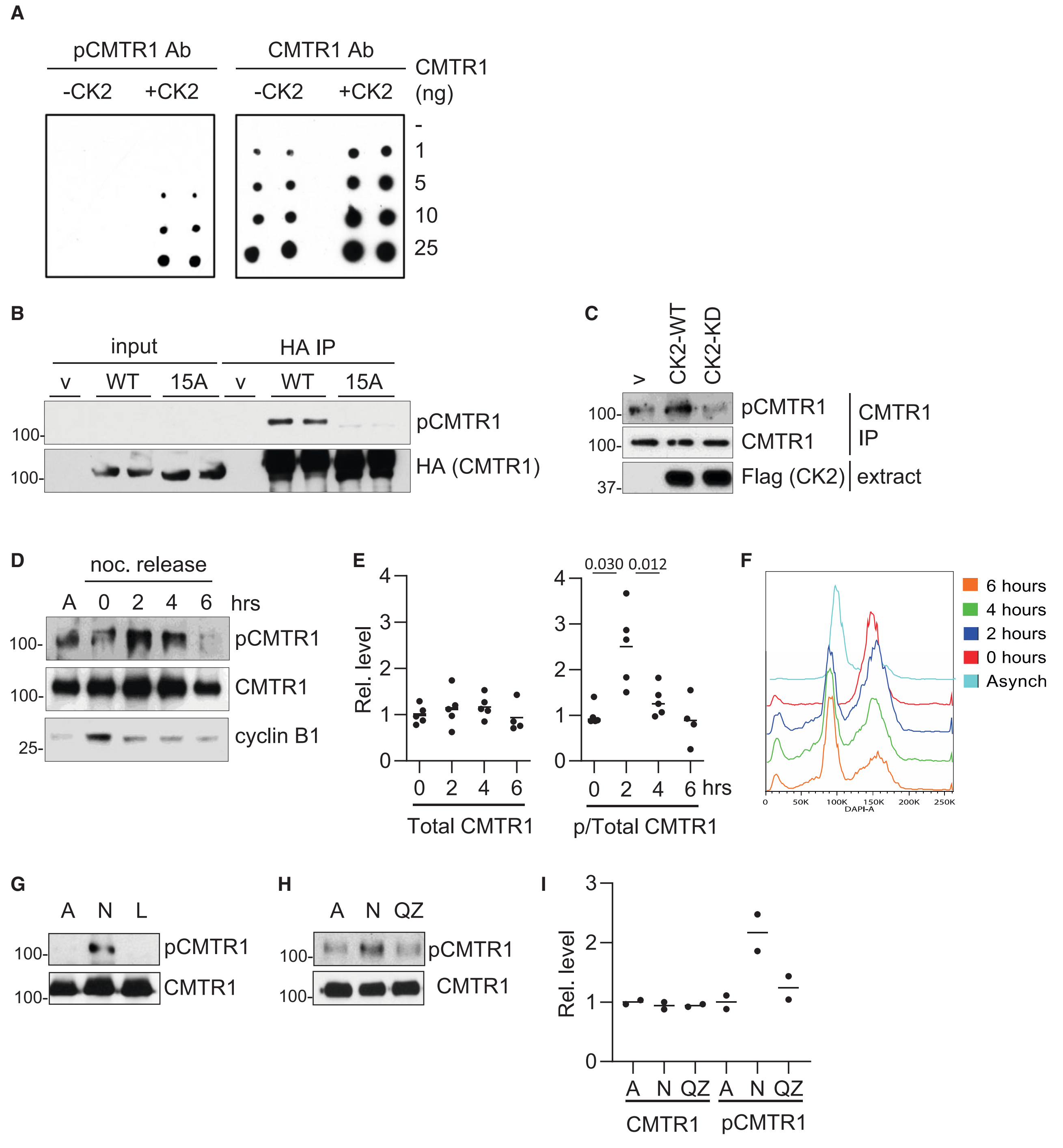

Figure 2. CMTR1 is phosphorylated by CK2 during G1 phase

(A) Recombinant CMTR1 was *in vitro* phosphorylated with CK2 and a titration was blotted onto PVDF (ng indicated). Blots were probed with pCMTR1 antibody or (pan) CMTR1 antibody.

(B) HA-CMTR1 WT, HA-CMTR1 15A, or empty vector (v) were transiently expressed in HeLa cells. HA-CMTR1 proteins were immunoprecipitated via the HA tag and analyzed by western blot.

(C) FLAG-CK2 WT, D156A (kinase dead [KD]), or empty vector (v) were transiently expressed in HeLa cells. Endogenous CMTR1 was immunoprecipitated and analyzed by western blot.

(D) HeLa cells were arrested in G2/M phase using nocodazole and released into the cell cycle by replacement with fresh medium. CMTR1 was immunoprecipitated over a time course of nocodazole release or from asynchronous cells (A) and analyzed by western blot for phospho-CMTR1 and total CMTR1 (representative shown). Cyclin B expression was analyzed.

(legend continued on next page)

CMTR1 did not significantly impact methyltransferase activity (Figure S3A). Similarly, using lambda phosphatase to reduce the phosphorylation of HA-CMTR1 immunoprecipitated from cells did not impact methyltransferase activity (Figures 2G and S3B). CMTR1 function can be influenced by interaction with the helicase DHX15.[18,19] An equivalent quantity of DHX15 was found in HA-CMTR1 WT and 15A complexes immunoprecipitated from HeLa cells, indicating that phosphorylation of CMTR1 does not influence this interaction (Figure S3C). As a control, the CMTR1 G-Patch 3L/A mutant had reduced interaction with DHX15[18] (Figure S3C). The CMTR1 P-Patch is adjacent to the NLS (Figure 1A).[18,32] To investigate whether CMTR1 phosphorylation impacts the cellular localization of the protein, GFP-CMTR1 WT and 15A were transfected into HeLa cells (Figure S4). As observed previously, GFP-CMTR1 WT has a predominantly diffuse nuclear localization, and GFP-CMTR1 15A has an equivalent nuclear localization.[18] As controls, GFP-CMTR1 25-831 and GFP-CMTR1 4/K/E, mutants with a deleted or mutated NLS, were predominantly cytoplasmic.[18]

CMTR1 is recruited to nascent RNA caps by an interaction of the WW domain with the RNA Pol II CTD phosphorylated on S5[14,18] and by an interaction of the GT-like domain with RNA Pol II PBP7.[24] To investigate whether the interaction of the RNA Pol II CTD with CMTR1 is influenced by phosphorylation, HA-CMTR1 WT, 15A, and vector control were expressed in MEFs (mouse embryonic fibroblasts) and HeLa cells (Figures 3A and 3B, respectively). HA-CMTR1 WT was immunoprecipitated from cell extracts in a complex with RNA Pol II S5P and S2P of the CTD (Figures 3A and 3B). The phospho-defective CMTR1 15A mutant had significantly reduced interaction with the CTD, consistent with CMTR1 phosphorylation promoting or permitting this interaction. To validate the role of CK2 in the CMTR1-RNA Pol II interaction, CK2 WT and KD were transiently expressed in HeLa cells (Figure 3C). Expression of CK2 WT, but not CK2 KD, resulted in increased CMTR1 phosphorylation and increased interaction with RNA Pol II (Figure 3C).

To investigate whether CK2 phosphorylation of CMTR1 directly influences the interaction with the RNA Pol II CTD, an *in vitro* binding assay was performed. As observed previously, recombinant CMTR1 interacted directly with the CTD peptide, with enhanced binding to the S5P CTD peptide (Figure 3D).[18] Phosphorylation of recombinant CMTR1 with CK2 increased its interaction with the RNA Pol II CTD (Figures 3E and 3F).

Intramolecular interactions of CMTR1 are controlled by P-Patch phosphorylation

CMTR1 recruitment to the RNA Pol II CTD requires the CMTR1 WW domain.[18] Here, we observe that phosphorylation of the CMTR1 P-Patch influences the RNA Pol II-CMTR1 interaction (Figure 3). Since phosphorylation of the CMTR1 N-terminal P-Patch influences an interaction of the C-terminal WW domain with RNA Pol II, this indicates a phosphorylation-induced change in conformation or an intramolecular interaction of CMTR1. To investigate the interactions between the different domains of CMTR1 a series of HA-CMTR1 N-terminal deletions and GFP-CMTR1 C-terminal deletions were made and co-expressed in HEK293 cells (Figure 4A). In co-immunoprecipitation experiments, GFP-CMTR1ΔWW (GFP-ΔWW) interacted with HA-CMTR1-WW (lane 9, Figure 4B) or HA-CMTR1-GT-WW (lane 9, Figure 4C). This revealed an interaction between the CMTR1 WW domain and another part of CMTR1. The more extensive deletion mutant GFP-CMTR1 1-143 (GFP-1-143) failed to interact with HA-CMTR1-WW (lane 8, Figure 4B) or HA-CMTR1-GT-WW (lane 8, Figure 4C), indicating that the CMTR1 RFM-GT-like (Rossman-fold methyltransferase-guanylyltransferase-like) domain is required for the interaction with the CMTR1 WW domain. These CMTR1 domain interactions are likely to reflect intramolecular interactions rather than *trans* interactions of two CMTR1 proteins since GFP-CMTR1 WT does not bind to any CMTR1 deletion mutant or another CMTR1 WT (Figures 4B and 4C, lane 10; data not shown). Consistent with this, in the AlphaFold2 prediction of the CMTR1 structure, the WW, GT-like, and RFM domains have multiple interactions[33,34] (Figure 4D).

The impact of CMTR1 intramolecular interactions on recruitment to RNA Pol II was investigated (Figures 4E and 4F). As published previously,[18] GFP-CMTR1ΔWW (WT or 15A) does not interact significantly with RNA Pol II (lanes 8 and 9, Figure 4E). When HA-CMTR1-GT-WW (HA-GT-WW) is expressed with GFP-CMTR1ΔWW (GFP-WT-ΔWW), they form a complex, permitting GFP-CMTR1ΔWW to bind to RNA Pol II (Figure 4E, lane 11). GFP-CMTR1ΔWW with 15A mutations (GFP-15A-ΔWW) also interacts with HA-GT-WW, but this complex has reduced RNA Pol II binding (Figure 4E, lane 12). These data are consistent with the CMTR1 P-Patch interacting with the CMTR1 CTD and this interaction promoting binding to RNA Pol II in a phospho-dependent manner. In the AlphaFold2-predicted structure, the P-Patch is a disordered region, which may contact the rest of CMTR1 at multiple points, enhancing the intramolecular interaction (Figure 4D).

Evidence supporting that the phosphorylated P-Patch promotes CMTR1-RNA Pol II interactions comes from experiments in which HA-CMTR1-GT-WW (HA-GT-WW) was expressed in cells and immunoprecipitated with RNA Pol II CTD S5P (Figure 4F, lane 7), consistent with the RNA Pol II CTD-CMTR1 WW domain interaction.[18] Although GFP-CMTR1ΔWW (GFP-WT-ΔWW) has a weak affinity for RNA Pol II, expression of it increases the interaction of HA-CMTR1-GT-WW and RNA Pol II (Figure 4D, compare lanes 8 and 9). GFP-CMTR1ΔWW 15A (GFP-15A-ΔWW) also interacts with HA-CMTR1-GT-WW

(E) pCMTR1/CMTR1 and total CMTR1 were quantitated. Dots indicate data for 4 or 5 independent experiments. Line indicates the average. Student's t test was performed, and *p* values are stated.
(F) Cell cycle progression in (D) was analyzed by flow cytometry using DAPI DNA stain. The proportion of cells in each stage of the cell cycle is indicated.
(G) Cells released from nocodazole block for 2 h were untreated (N), treated with lambda phosphatase (L), or asynchronous (A).
(H) As in (G) except cells were treated with quinalizarin (QZ).
(I) Detection of pCMTR1/CMTR1 and total CMTR1 was quantitated for 2 independent experiments. Dots indicate data, and line indicates the average.
See also Figures S3 and S4.

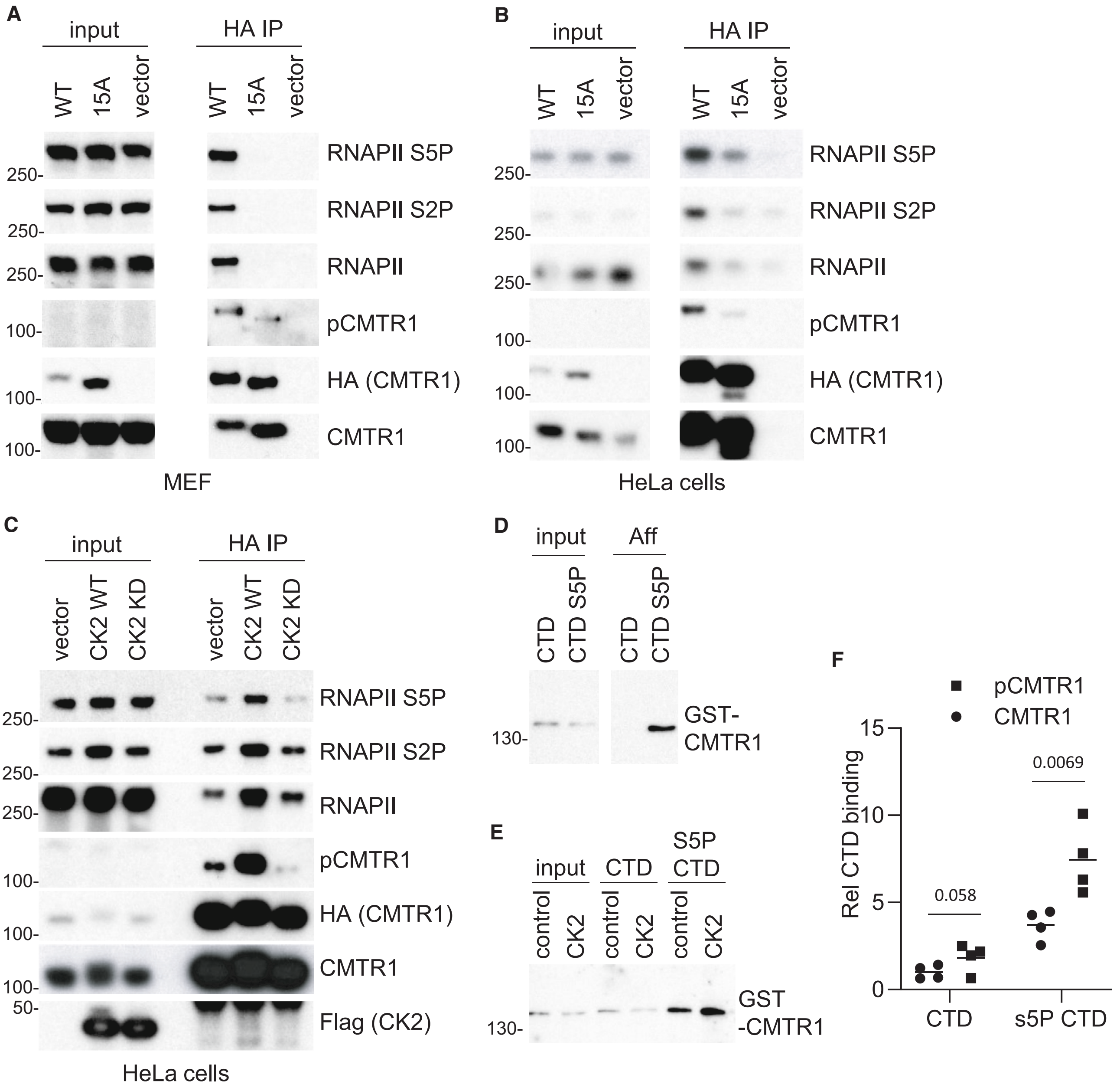

Figure 3. CK2 phosphorylation of CMTR1 increased interaction with RNA Pol II

(A and B) HA-CMTR1 WT, 15A, or vector control. Dots indicate data were transiently expressed in (A) MEFs or (B) HeLa cells. HA-CMTR1 WT or 15A was immunoprecipitated from cell extracts via the HA tag. Western blots were performed on input material and immunoprecipitates (IPs).

(C) HA-CMTR1 was transiently co-expressed in HeLa cells with CK2 WT, CK2 KD, or vector control. Western blots were performed on input material and HA-CMTR1 IPs for the antigens indicated.

(D) Recombinant GST-CMTR1 was incubated with biotinylated CTD peptide, unphosphorylated (CTD), or phosphorylated on serine-5 (CTD S5P). CMTR1 was detected by western blot in inputs and streptavidin pull-downs (Aff).

(E) As in (D) except GST-CMTR1 was *in vitro* phosphorylated by incubation with CK2 prior to CTD and CTD-S5P pull-downs.

(F) GST-CMTR1 binding to CTD was quantitated for 4 independent experiments Dots indicate data, and line indicates the average. Student's t test was performed, and *p* values are stated.

(HA-GT-WW) but does not increase the interaction with RNA Pol II (Figure 4F, compare lanes 8 and 10). Thus, P-Patch phosphorylation (even on a distinct peptide) positively influences the interaction of the RNA Pol II CTD and CMTR1.

CMTR1 phosphorylation increases RNA cap formation and cell proliferation

To investigate the impact of CMTR1 phosphorylation in cells, we utilized MEFs in which the *Cmtr1* gene is floxed in exon 3,

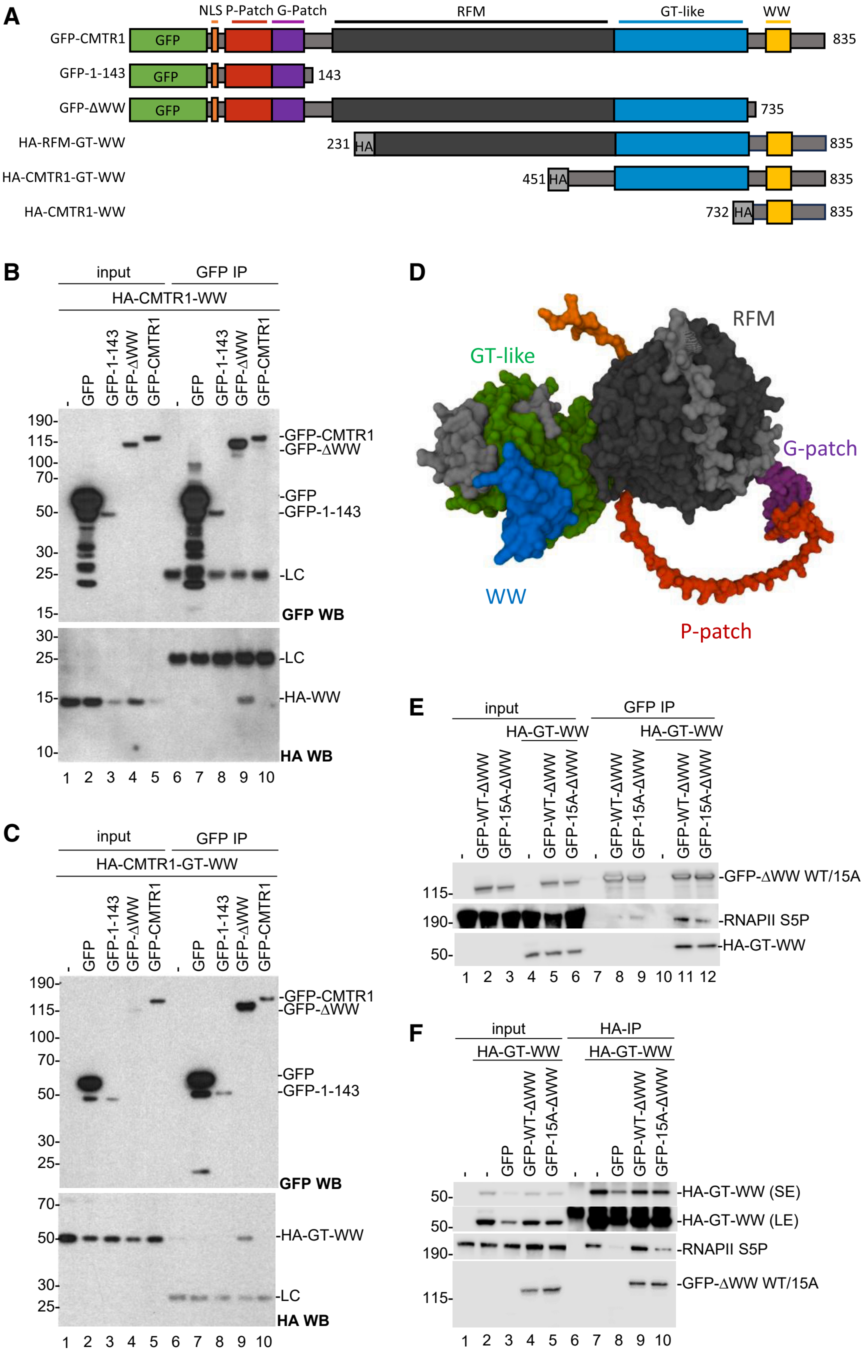

Figure 4. Intramolecular interactions of CMTR1

(A) Diagram of CMTR1 mutants used.

(B) HA-CMTR1-WW was transiently co-expressed with the GFP-CMTR1 WT and mutants indicated above blots in HeLa cells. GFP-CMTR1 proteins were immunoprecipitated using GFP nanobodies and western blots performed to detect HA-WW (anti-HA antibody) and GFP-tagged protein (anti-GFP antibodies).

(C) As in (B) except HA-CMTR1-GT-WW was co-expressed with GFP-tagged CMTR1 mutants.

(D) The predicted structure of CMTR1 by AlphaFold2. The domains of interest are indicated.

(E and F) HA-CMTR1-GT-WW was expressed with GFP-CMTR1 WT, 15A, or GFP alone. (E) GFP-CMTR1 proteins immunoprecipitated with nanobodies. (F) HA-GT-WW was immunoprecipitated using anti-HA antibodies. RNA Pol II S5P and other antigens were detected by western blot.

resulting in gene deletion upon expression of Cre recombinase (Figure S5). In these cells, HA-CMTR1 WT and 15A were expressed following retroviral infection (at a level lower than endogenous CMTR1), and subsequently, the endogenous *Cmtr1* gene was deleted (Figure S5C). Using this method, we avoided cell adaptation to long-term *Cmtr1* gene deletion, which we observed previously (CMTR1 15A expression increases relative to WT over time in *Cmtr1*$^{-/-}$ cells; data not shown). The impact of CMTR1 15A on RNA cap formation was analyzed by CAP-MAP (cap analysis protocol with minimal analyte processing) MS.[35] Consistent with CMTR1 being required for N1 2′-*O*-Me (O-2 methylation of the first transcribed nucleotide ribose), *Cmtr1* deletion resulted in a reduction in the N1 O-2-Me-containing mature RNA caps ^{m7}Gppp^{m6}A$_m$, ^{m7}GpppA$_m$, and ^{m7}GpppG$_m$ and an increase in the incomplete caps ^{m7}Gppp^{m6}A, ^{m7}GpppA, and ^{m7}GpppG in comparison to cells expressing CMTR1 WT (Figure 5A). Expression of CMTR1 15A only partially rescued the abundance of mature caps, consistent with CMTR1 phosphorylation promoting cellular RNA cap O-2 methylation.

Phospho-defective mutation 15A reduces CMTR1-dependent gene expression

CMTR1 and its product N1 2′-*O*-Me have roles in gene expression, with the target genes determined by the cell lineage.[8,13,14,17,18,20,21] RNA sequencing analysis was carried out on log-phase MEFs expressing HA-CMTR1 WT, 15A, or vector control, in which the endogenous *Cmtr1* gene was deleted (Figure S5). In previous studies, CMTR1 repression had been demonstrated to impact RNA levels.[14,15] In RNA sequencing analysis, 12,516 RNAs (transcripts mapping to single genes) were detected that had more than one count per million reads in more than 3 samples (Table S1). Expression of HA-CMTR1 WT resulted in significantly increased levels of 1,521 RNAs and significantly decreased levels of 1,334 RNAs (Figures 5B and 5C). Expression of HA-CMTR1 15A also resulted in altered RNA levels but to a lesser extent than HA-CMTR1 WT, consistent with CMTR1 phosphorylation being required for RNA Pol II binding and RNA capping. 797 RNAs were significantly

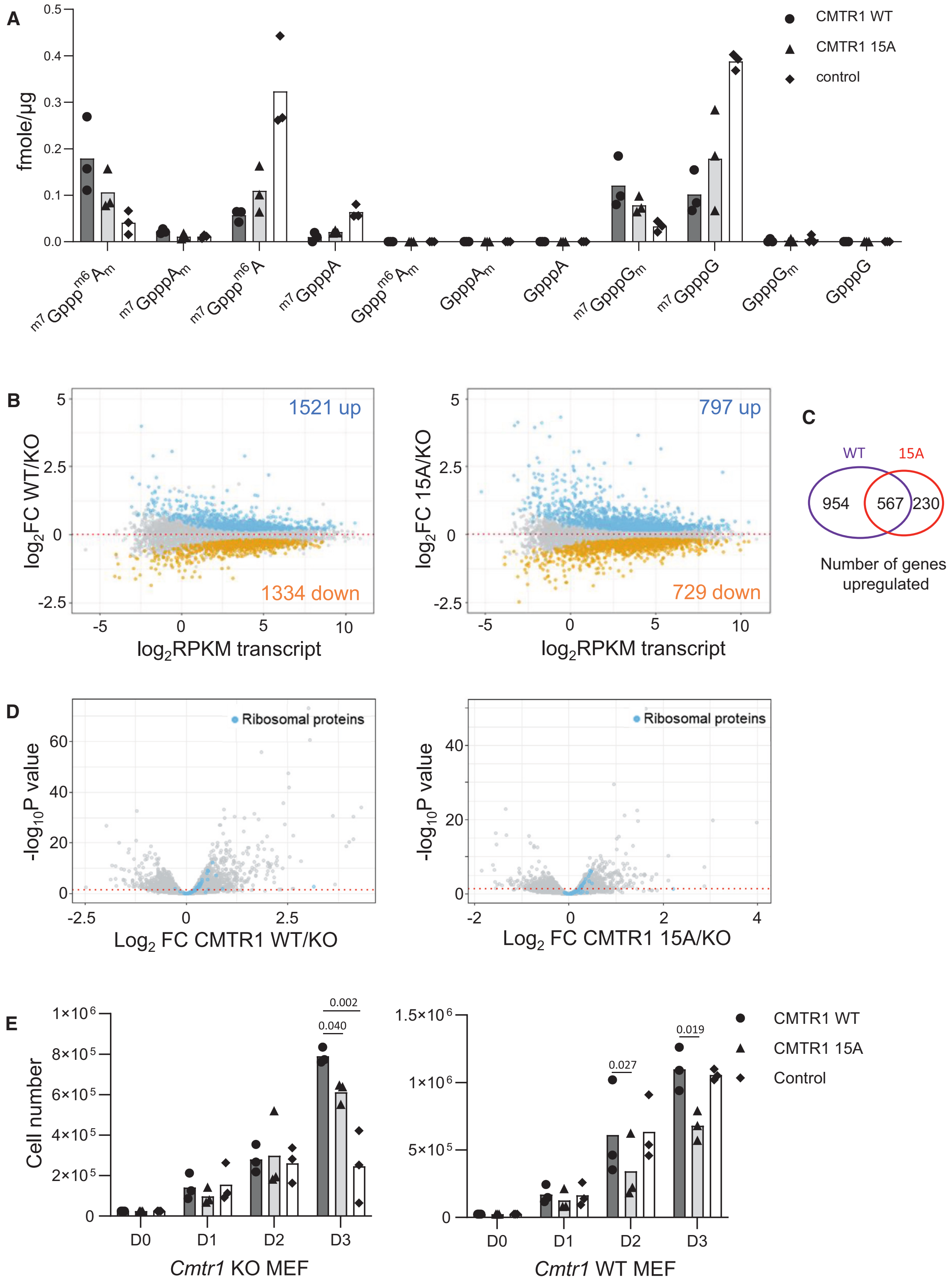

(legend on next page)

increased in response to the expression of HA-CMTR1 15A, and 729 RNAs were significantly reduced (Figures 5B and 5C). 567 of the same RNAs were increased in HA-CMTR1 WT- and 15A-expressing cells; RNAs upregulated in response to CMTR1 15A were largely a subset of those upregulated in response to the WT protein (Figure 5C). Furthermore, HA-CMTR1 WT expression resulted in higher increases in RNA levels compared to CMTR1 15A. Of the top 100 RNAs increased in response to CMTR1 WT, the average LFC (log fold change) was 1.96, whereas for the top 100 RNAs that increased in response to CMTR1 15A, the average LFC was 1.53 (Table S1).

Gene Ontology term analysis revealed that genes upregulated by both WT and 15A included genes involved in RNA translation, consistent with previous studies (Figure S6).[14,15] One of the most CMTR1-dependent gene families in embryonic stem cells is the ribosomal protein genes, correlating with high RNA Pol II-gene binding.[14] Ribosomal protein gene transcripts were also induced in response to CMTR1 WT and 15A, indicating the conservation of their CMTR1 dependency across cell types (Figure 5D; Table S1). CMTR1 WT induced ribosomal protein gene transcripts more that the 15A mutant, consistent with increased RNA Pol II binding.[14] Consistent with reduced gene expression, deletion of the *Cmtr1* gene in MEFs resulted in reduced cell proliferation (Figure 5E). Expression of CMTR1 WT rescued this defect more than 15A (Figure 5E, left). In cells expressing endogenous CMTR1, expression of CMTR1 15A acted as a dominant negative, reducing cell proliferation (Figure 5E, right).

CMTR1 phosphorylation is required for the interferon response

CMTR1 has previously been demonstrated to be required for the expression of ISGs.[13] Here, we observed that the ISGs IFIT1, IFIT3, IFIH1, ISG15, and DHX58 are upregulated in response to interferon addition in CMTR1 WT-expressing *Cmtr1*$^{-/-}$ MEFs (Figure 6A). Interferon-induced expression of IFIT1, IFIT3, IFIH1, and ISG15 was significantly reduced in MEFs expressing CMTR1 15A (Figure 6A). Consistent with these observations, interferon-induced expression of IFIT3 and ISG15 proteins was delayed in *Cmtr1*$^{-/-}$ MEFs compared to those expressing HA-CMTR1 WT (Figure 6B). This was most apparent at the 4 h time point. Expression of HA-CMTR1 15A did not rescue this defect. PolyI:C (polyinosinic:polycytidylic acid) mimics RNA species generated during viral replication and is sensed by TLR3, MDA5, and RIG-I, resulting in interferon expression and other impacts.[36] Transfection of MEFs with polyI:C resulted in IFIT1 and IFIT3 expression in cells expressing CMTR1 WT, and this was attenuated in cells expressing CMTR1 15A (Figure 6C). Since the induction of the ISGs is dependent on an intact P-Patch, this implies that CK2 phosphorylation of CMTR1 is important in the interferon response. We used the highly selective CK2 competitive inhibitor quinalizarin to further investigate the role of CK2 in the interferon response.[31] Pretreatment of MEFs with quinalizarin reduced the interferon-dependent induction of ISGs (Figure 6D) (we note that many proteins involved in the response to interferon are phosphorylated by CK2, and therefore quinalizarin is likely to have impacts beyond CMTR1 phosphorylation[26]).

CMTR1 phosphorylation is required for influenza infection

IAV is a negative-sense, single-stranded RNA virus that is dependent on the removal of host cell RNA caps via endonucleases for ligation to viral transcripts, a process known as cap snatching.[37] Influenza virus infection of A549 cells is dependent on CMTR1.[38] We investigated whether the defect in RNA cap production in cells expressing CMTR1 15A impacts influenza infection. A mouse-adapted PR8 IAV strain expressing a fluorescent mCherry reporter fused to the open reading frame of the viral NS1 gene was used to infect MEFs, with infected cells detected by mCherry protein expression at 17 and 24 h post-infection (Figure 6E).[39] Deletion of *Cmtr1* resulted in a significant reduction in the number of infected cells, and this could be rescued by the expression of CMTR1 WT. The expression of CMTR1 15A could partially rescue the defect, but the number of infected cells was significantly lower than that in cells expressing CMTR1 WT. CMTR1 phosphorylation could also be linked to viral protein detection (Figure 6F). Of the cells positive for viral infection, the geometric mean fluorescence intensity for NS1 expression was significantly higher in cells expressing CMTR1 WT in a *Cmtr1*$^{-/-}$ background compared to CMTR1 15A. Therefore, although CMTR1 phosphorylation is required for the interferon response, which can suppress aspects of viral infection, in the context of IAV infection, CMTR1 phosphorylation acts as a pro-viral factor. This can be potentially attributed to the dependence of IAV on cap-snatching mechanisms for efficient expression of viral mRNAs alongside its ability to suppress the interferon response.[40] In addition, members of the interferon-stimulated IFIT family are pro-viral factors that promote the translation of IAV mRNA[41,42]; therefore, the CMTR1 phosphorylation-dependent expression of IFIT mRNAs may also contribute to efficient infection.

Figure 5. CMTR1 phosphorylation is required for RNA cap methylation and gene expression

MEF lines were created to express HA-CMTR1 WT, 15A, and vector control. Cre recombinase was expressed to delete the *Cmtr1* gene.

(A) Relative abundance of cap structures in *Cmtr1* knockout (KO) MEFs expressing HA-CMTR1 WT, 15A, or vector control. Data presented are from three independent experiments, and bar indicates the average.

(B) MA plots of transcript levels (log_2RPKM [reads per kilobase per million mapped reads]) and log_2 fold change of CMTR1 WT vs. KO (left) and CMTR1 15A vs. KO (right). Genes significantly down-/up-regulated (EdgeR exactTest, false discovery rate [FDR]-adjusted $p < 0.05$) are highlighted.

(C) Venn diagram of numbers of genes increased by expression of CMTR1 WT and 15A, defined as those gene transcripts significantly up-regulated relative to *Cmtr1* KO.

(D) Volcano plots indicating the relationship between log_2FC and $-log_{10}$ FDR-adjusted p value for CMTR1 WT vs. KO (left) and CMTR1 15A vs. KO (right). Ribosomal protein genes (79 genes) are highlighted.

(E) MEF lines with and without Cre-directed *Cmtr1* deletion. MEFs were plated and counted each day. Data shown are for 3 experiments, and bar indicates the average. Student's t test was performed, and p values are stated.

See also Figures S5 and S6.

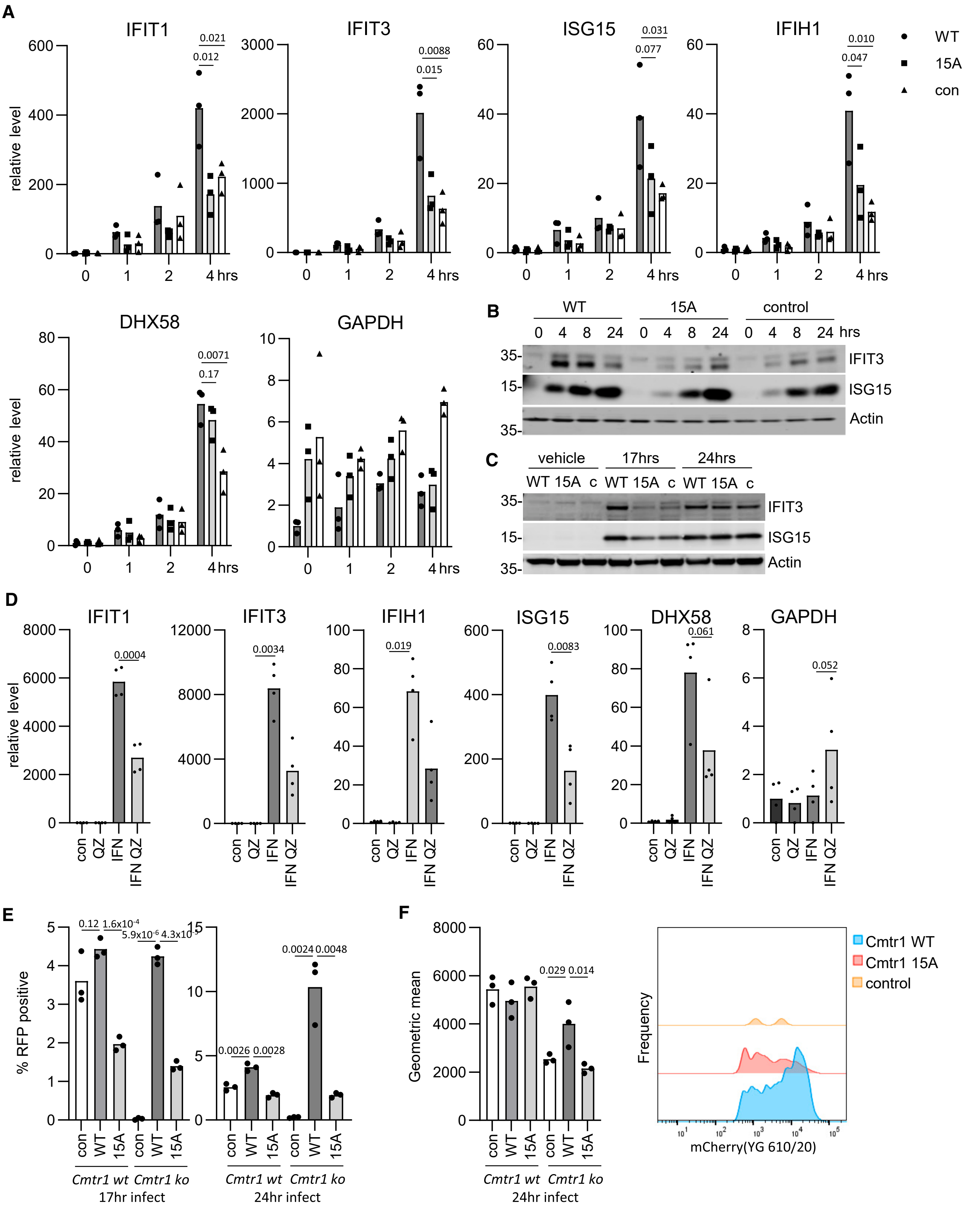

(legend on next page)

CellPress
OPEN ACCESS

DISCUSSION

RNA Pol II transcripts are methylated at the O-2 position of first transcribed nucleotide ribose (N1 2′-*O*-Me), a modification that is part of the RNA cap.[2,5] Here, we report that CMTR1, the N1 2′-*O*-Me cap methyltransferase, is regulated by CK2-dependent phosphorylation on an N-terminal domain, which we named the P-Patch. Multiple serines and threonines in the P-Patch (CMTR1 amino acids 28–89) are substrates for CK2, although this domain may be phosphorylated by other kinases too. CMTR1 is recruited to the hypomethylated RNA cap by interactions with the RNA Pol II complex at the initiation of transcription.[14,18,24] When the P-Patch is phosphorylated, the affinity of CMTR1 for the RNA Pol II CTD increases. Consistent with enhanced recruitment to RNA Pol II, we report that phosphorylation of CMTR1 is required for cap ribose-O-2 methylation and the expression of a subsets of RNAs, including ribosomal protein gene transcripts and ISGs. Phosphorylation of CMTR1 is also required for production of influenza virus, which cap snatches, taking the RNA cap from host cell transcripts for the priming of viral transcription and evasion of host immunity.[43] CK2 is a potent kinase that directs cellular functions.[25–28] CK2 can phosphorylate specific amino acids on substrates or deposit patches of phosphorylation, with impacts on protein expression, substrate interactions, localization, and activity.[44–46] CK2 or CK2-dependent phosphorylation is deregulated in many cancers, neurological conditions, and immune disorders. Inhibiting CK2 has biological impacts in specific disease areas, and as a result, CK2-targeting strategies are being investigated.[27,28,45,46] Here, we demonstrate that CK2-dependent phosphorylation of CMTR1 is required for efficient cell proliferation, the interferon response, and influenza virus production.

CMTR1 is phosphorylated during G1 phase of the cell cycle and promotes cell proliferation. Phosphorylation of another RNA cap methyltransferase, the cap guanosine N-7 methyltransferase (RNMT, RNA guanine-7 methyltransferase), also promotes cell proliferation.[30] RNMT is phosphorylated by CDK1-cyclin B during late G2/M phase through to G1 phase, a modification that increases catalytic activity.[30] Being present during G1 phase, RNMT phosphorylation and CMTR1 phosphorylation enhance RNA cap formation when most transcripts are being synthesized. CK2 is not a cell cycle-regulated kinase, but it does interact with many proteins involved in chromatin dynamics and transcription including RNA Pol II.[47] CMTR1 may be phosphorylated by proximal CK2 when it is recruited to the transcribing RNA Pol II during G1 phase. Of note, CK2 also modulates the action of signaling and mechanistic proteins involved in transcription and translation.[27,47]

CMTR1 makes at least 2 contacts with the RNA Pol II complex: the CMTR1 C-terminal WW domain interacts with the RNA Pol II CTD and the adjacent CMTR1 GT-like domain interacts with the RBP7 subunit.[18,24] How does phosphorylation of the P-Patch at the N terminus of CMTR1 impact the interaction of the C-terminal WW domain with the RNA Pol II CTD? We confirmed that the WW domain is required for interaction with RNA Pol II. Additionally, we report that other regions of CMTR1 can interact with the WW domain and indirectly promote interaction with the polymerase. This stimulatory impact of CMTR1 is dependent on P-Patch phosphorylation; mutation of the P-Patch phospho-sites nullifies the stimulatory impact on the WW domain-RNA Pol II interaction. Structural information about the P-Patch is not available; based on AlphaFold predictions, it is likely to be disordered. We speculate that the phosphorylated P-Patch may interact with a positively charged patch in the WW domain (or proximal region), resulting in intramolecular alterations that support the CMTR1 and RNA Pol II CTD interaction.

The gene specificity of CMTR1 in altering RNA levels is important to understand because it dictates its biological impact. CMTR1 has direct and indirect roles in transcription and RNA stability, both of which will impact RNA levels.[14,15] Current information about the factors dictating gene specificity is limited: high levels of RNA Pol II and CMTR1 gene binding correlate loosely with a response of genes to CMTR1 inhibition, but other factors are indicated, and differential affinity for 5′ RNA sequences may also have a role.[14,15,20] Which RNAs are CMTR1 dependent varies with cell lineage.[1,8,14,16–21] In MEFs, expression of the phospho-defective CMTR1 15A mutant regulates the expression of a similar set of RNAs as the WT protein but simply regulates them less effectively. Approximately half as many RNAs are upregulated to threshold levels by CMTR1 15A, compared to WT, in log-phase conditions. As in embryonic stem cells, major CMTR1 targets include the ribosomal protein gene transcripts. Ribosomal protein genes have some of the highest levels of RNA Pol II and CMTR1 bound to the transcription start site.[14,15]

CMTR1 phosphorylation also has a role in the interferon response; it is an ISG (interferon-stimulated gene), which facilitates the expression of other ISGs.[13] Here, we demonstrate that ISG expression is dependent on CMTR1 phosphorylation. In liver hepatoma cells and human monocyte cell lines, CMTR1 has little impact on ISG RNA levels but does impact on protein levels,[13] whereas we report that in MEFs, CMTR1 impacts the RNA levels of ISGs. The cap interacts with multiple complexes

Figure 6. Induction of interferon-stimulated genes is dependent on CMTR1 P-Patch phosphorylation

(A) *Cmtr1* KO MEF lines expressing HA-CMTR1 WT, 15A, and vector control were incubated in 400 U/mL interferon for 1, 2, and 4 h. RNA was harvested and RNAs detected by PCR. Data are from 3 independent experiments, and bar indicates the average. Student's t test was performed, and *p* values are indicated.

(B and C) MEFs were (B) incubated with 400 U/mL interferons for 0, 4, 8, and 24 h and (C) transfected with 5 μg/mL polyI:C for 17 and 24 h. IFIT3 and ISG15 proteins were analyzed by western blot. Actin was used as a loading control.

(D) WT MEFs were incubated with 10 μM QZ for 0.5–3 h and 400 U/mL interferon for a subsequent 4 h. RT-PCR was performed as above. Data are from 4 independent experiments. Student's t test was performed, and *p* values are indicated.

(E) *Cmtr1* WT and KO MEF lines expressing HA-CMTR1 WT, 15A, and vector control (con) were incubated with PR8 ColorFlu expressing a mCherry reporter gene. Cells producing virus were detected by fluorescence-activated cell sorting at 17 and 24 h post-infection. Data are from 3 independent wells. Student's t test was performed, and *p* values are indicated.

(F) For mCherry-positive cells, the geometric mean of red fluorescence was reported. A sample histogram is presented for $Cmtr1^{-/-}$ MEFs expressing HA-CMTR1 WT, 15A, and vector control, normalized to the mode.

involved in RNA processing and degradation; which processes are most dependent on ribose O-2 methylation will depend on the cellular context, including the levels of the different cap-binding proteins. In addition, CMTR1 can have impacts on transcription, either by the RNA cap protecting transcripts from degradation or by a more direct impact on transcription.[15] Although CMTR1 is an ISG itself, in MEFs, the IFN-induced increase in CMTR1 protein occurs after 12–24 h and therefore will only contribute to the interferon response at these later time points.

The impact of CMTR1 phosphorylation on the interferon response is likely to protect cells from a range of infections. However, in the context of influenza infection, inhibition of CMTR1 phosphorylation reduced the expression of the viral protein NS1 and the number of infected cells. Influenza viruses acquire RNA caps for their transcripts by removing them from cellular transcripts (cap snatching) and are therefore dependent on host capped mRNA abundance for efficient transcription and subsequent translation of viral mRNAs.[38,48,49] Other viruses that do not require cellular caps may be inhibited by CMTR1-dependent regulation of the interferon response. Unusually, influenza viruses also require IFIT family members, known ISGs, for efficient infection.[41,42] Therefore, CMTR1 phosphorylation-dependent expression of IFIT genes and the requirement for CMTR1 phosphorylation-dependent cellular caps may both contribute to efficient IAV infection.

In conclusion, we present a mechanism, CK2-dependent CMTR1 phosphorylation, that enhances mRNA capping during critical transcriptional bursts, such as cell cycle progression or immune defense.

Limitations of the study

Here, we demonstrate that the pleiotropic kinase CK2 phosphorylates the RNA cap methyltransferase CMTR1, resulting in a conformational change that promotes interaction with RNA Pol II and RNA cap methylation. Our data demonstrate that CK2 is the predominant CMTR1 kinase in HeLa cells; however, it is possible that other kinases phosphorylate the P-Patch. Indeed, proteins that are phosphorylated at multiple sites are often substrates for multiple kinases, with the kinases acting independently or in a hierarchical manner. Initial phosphorylation can often generate recognition motifs for subsequent kinases to be recruited and act on the same region. The amino acid sequence of the CMTR1 P-Patch may be a substrate for alternative acidophilic kinases, including CK1, GSK3b, Plk, or Fam20C.

STAR★METHODS

Detailed methods are provided in the online version of this paper and include the following:

- KEY RESOURCES TABLE
- RESOURCE AVAILABILITY
  - Lead contact
  - Materials availability
  - Data and code availability
- EXPERIMENTAL MODEL AND STUDY PARTICIPANT DETAILS
  - Animals
  - Cell lines
  - Primary cell cultures
- METHOD DETAILS
  - Cell transfection and infection
  - MEF immortalisation and Cre recombinase-based CMTR1 deletion
  - Cell synchronisation
  - Colorflu infection and FACS detection
  - Mass spectrometry
  - *In vitro* kinase assay
  - Molecular biology
  - Cell lysis and Co-immunoprecipitation
  - Antibody production
  - Dot blot
  - Western Blot and quantification
  - RNA sequencing analysis
- QUANTIFICATION AND STATISTICAL ANALYSIS

SUPPLEMENTAL INFORMATION

Supplemental information can be found online at https://doi.org/10.1016/j.celrep.2024.114405.

ACKNOWLEDGMENTS

We would like to thank Gopal Sapkota, University of Dundee; Rick Randall, University of St Andrews; Ed Hutchinson and Alfredo Castello, University of Glasgow; and the Cowling lab for advice, reagents, and technical assistance. We would like to thank the MRC Protein Phosphorylation and Ubiquitination Unit, Division of Signal Transduction Therapies, and University of Dundee TAGC for technical assistance. We thank the Core Services and Advanced Technologies at the Cancer Research UK Scotland Institute (A31287), with particular thanks to the Beatson Advanced Imaging Resource. The manuscript was critically reviewed by Catherine Winchester (CRUK Scotland Institute). This work was supported by Cancer Research UK core grant number A17196/A31287 to the CRUK Scotland Institute and CTRQQR-2021\100006 to the CRUK Scotland Centre. Research was funded by European Research Council Award 769080 TCAPS, Medical Research Council Senior Fellowship MR/K024213/1, a Lister Research Prize Fellowship, a Wellcome Trust PhD studentship 097462/Z/11/Z, Royal Society Wolfson Research Merit Award WRM\R1\180008, Wellcome Trust Investigator Award 219416/A/19/Z, and Wellcome Trust GRE Centre Award 097945/Z/11/Z.

AUTHOR CONTRIBUTIONS

Conceptualization, F.I.V. and V.H.C.; data curation, all authors; analysis, all authors; investigation, all authors; writing, all authors; supervision, V.H.C.

DECLARATION OF INTERESTS

The authors declare no competing interests.

Received: March 13, 2024
Revised: May 12, 2024
Accepted: June 11, 2024
Published: June 25, 2024

REFERENCES

1. Dohnalkova, M., Krasnykov, K., Mendel, M., Li, L., Panasenko, O., Fleury-Olela, F., Vågbø, C.B., Homolka, D., and Pillai, R.S. (2023). Essential roles of RNA cap-proximal ribose methylation in mammalian embryonic development and fertility. Cell Rep. *42*, 112786. https://doi.org/10.1016/j.celrep.2023.112786.
2. Inesta-Vaquera, F., and Cowling, V.H. (2017). Regulation and function of CMTR1-dependent mRNA cap methylation. Wiley Interdiscip. Rev. RNA *8*, e1450. https://doi.org/10.1002/wrna.1450.
3. Belanger, F., Stepinski, J., Darzynkiewicz, E., and Pelletier, J. (2010). Characterization of hMTr1, a human Cap1 2'-O-ribose methyltransferase. J. Biol. Chem. *285*, 33037–33044. https://doi.org/10.1074/jbc.M110.155283.

CellPress
OPEN ACCESS

4. Smietanski, M., Werner, M., Purta, E., Kaminska, K.H., Stepinski, J., Darzynkiewicz, E., Nowotny, M., and Bujnicki, J.M. (2014). Structural analysis of human 2'-O-ribose methyltransferases involved in mRNA cap structure formation. Nat. Commun. *5*, 3004. https://doi.org/10.1038/ncomms4004.

5. Pelletier, J., Schmeing, T.M., and Sonenberg, N. (2021). The multifaceted eukaryotic cap structure. Wiley Interdiscip. Rev. RNA *12*, e1636. https://doi.org/10.1002/wrna.1636.

6. Kruse, S., Zhong, S., Bodi, Z., Button, J., Alcocer, M.J.C., Hayes, C.J., and Fray, R. (2011). A novel synthesis and detection method for cap-associated adenosine modifications in mouse mRNA. Sci. Rep. *1*, 126. https://doi.org/10.1038/srep00126.

7. Grudzien-Nogalska, E., and Kiledjian, M. (2017). New insights into decapping enzymes and selective mRNA decay. Wiley Interdiscip. Rev. RNA *8*. https://doi.org/10.1002/wrna.1379.

8. Sikorski, P.J., Warminski, M., Kubacka, D., Ratajczak, T., Nowis, D., Kowalska, J., and Jemielity, J. (2020). The identity and methylation status of the first transcribed nucleotide in eukaryotic mRNA 5' cap modulates protein expression in living cells. Nucleic Acids Res. *48*, 1607–1626. https://doi.org/10.1093/nar/gkaa032.

9. Su, A.I., Pezacki, J.P., Wodicka, L., Brideau, A.D., Supekova, L., Thimme, R., Wieland, S., Bukh, J., Purcell, R.H., Schultz, P.G., and Chisari, F.V. (2002). Genomic analysis of the host response to hepatitis C virus infection. Proc. Natl. Acad. Sci. USA *99*, 15669–15674. https://doi.org/10.1073/pnas.202608199.

10. Geiss, G.K., Carter, V.S., He, Y., Kwieciszewski, B.K., Holzman, T., Korth, M.J., Lazaro, C.A., Fausto, N., Bumgarner, R.E., and Katze, M.G. (2003). Gene expression profiling of the cellular transcriptional network regulated by alpha/beta interferon and its partial attenuation by the hepatitis C virus nonstructural 5A protein. J. Virol. *77*, 6367–6375.

11. Guerra, S., López-Fernández, L.A., Pascual-Montano, A., Muñoz, M., Harshman, K., and Esteban, M. (2003). Cellular gene expression survey of vaccinia virus infection of human HeLa cells. J. Virol. *77*, 6493–6506.

12. Kato, A., Homma, T., Batchelor, J., Hashimoto, N., Imai, S., Wakiguchi, H., Saito, H., and Matsumoto, K. (2003). Interferon-alpha/beta receptor-mediated selective induction of a gene cluster by CpG oligodeoxynucleotide 2006. BMC Immunol. *4*, 8.

13. Williams, G.D., Gokhale, N.S., Snider, D.L., and Horner, S.M. (2020). The mRNA Cap 2'-O-Methyltransferase CMTR1 Regulates the Expression of Certain Interferon-Stimulated Genes. mSphere *5*, e00202. https://doi.org/10.1128/mSphere.00202-20.

14. Liang, S., Silva, J.C., Suska, O., Lukoszek, R., Almohammed, R., and Cowling, V.H. (2022). CMTR1 is recruited to transcription start sites and promotes ribosomal protein and histone gene expression in embryonic stem cells. Nucleic Acids Res. *50*, 2905–2922. https://doi.org/10.1093/nar/gkac122.

15. Liang, S., Almohammed, R., and Cowling, V.H. (2023). The RNA cap methyltransferases RNMT and CMTR1 co-ordinate gene expression during neural differentiation. Biochem. Soc. Trans. *51*, 1131–1141. https://doi.org/10.1042/BST20221154.

16. Kuge, H., Brownlee, G.G., Gershon, P.D., and Richter, J.D. (1998). Cap ribose methylation of c-mos mRNA stimulates translation and oocyte maturation in Xenopus laevis. Nucleic Acids Res. *26*, 3208–3214.

17. Lee, Y.L., Kung, F.C., Lin, C.H., and Huang, Y.S. (2020). CMTR1-Catalyzed 2 '-O-Ribose Methylation Controls Neuronal Development by Regulating Camk2 alpha Expression Independent of RIG-I Signaling. Cell Rep. *33*, 108269. https://doi.org/10.1016/j.celrep.2020.108269.

18. Inesta-Vaquera, F., Chaugule, V.K., Galloway, A., Chandler, L., Rojas-Fernandez, A., Weidlich, S., Peggie, M., and Cowling, V.H. (2018). DHX15 regulates CMTR1-dependent gene expression and cell proliferation. Life Sci. Alliance *1*, e201800092. https://doi.org/10.26508/lsa.201800092.

19. Toczydlowska-Socha, D., Zielinska, M.M., Kurkowska, M., Astha, Almeida, C.F., Stefaniak, F., Purta, E., and Bujnicki, J.M. (2018). Human RNA cap1 methyltransferase CMTr1 cooperates with RNA helicase DHX15 to modify RNAs with highly structured 5' termini. Philos. Trans. R. Soc. Lond. B Biol. Sci. *373*, 20180161. https://doi.org/10.1098/rstb.2018.0161.

20. Haussmann, I.U., Wu, Y., Nallasivan, M.P., Archer, N., Bodi, Z., Hebenstreit, D., Waddell, S., Fray, R., and Soller, M. (2022). CMTr cap-adjacent 2'-O-ribose mRNA methyltransferases are required for reward learning and mRNA localization to synapses. Nat. Commun. *13*, 1209. https://doi.org/10.1038/s41467-022-28549-5.

21. You, A.B., Yang, H., Lai, C.P., Lei, W., Yang, L., Lin, J.L., Liu, S.C., Ding, N., and Ye, F. (2023). CMTR1 promotes colorectal cancer cell growth and immune evasion by transcriptionally regulating STAT3. Cell Death Dis. *14*, 245. https://doi.org/10.1038/s41419-023-05767-3.

22. Meisel, J.D., Wiesenthal, P.P., Mootha, V.K., and Ruvkun, G. (2024). CMTR-1 RNA methyltransferase mutations activate widespread expression of a dopaminergic neuron-specific mitochondrial complex I gene. Curr. Biol. https://doi.org/10.1016/j.cub.2024.04.079.

23. Haline-Vaz, T., Silva, T.C.L., and Zanchin, N.I.T. (2008). The human interferon-regulated ISG95 protein interacts with RNA polymerase II and shows methyltransferase activity. Biochem. Biophys. Res. Commun. *372*, 719–724. https://doi.org/10.1016/j.bbrc.2008.05.137.

24. Garg, G., Dienemann, C., Farnung, L., Schwarz, J., Linden, A., Urlaub, H., and Cramer, P. (2023). Structural insights into human co-transcriptional capping. Mol. Cell *83*, 2464–2477.e5. https://doi.org/10.1016/j.molcel.2023.06.002.

25. St-Denis, N., Gabriel, M., Turowec, J.P., Gloor, G.B., Li, S.S.C., Gingras, A.C., and Litchfield, D.W. (2015). Systematic investigation of hierarchical phosphorylation by protein kinase CK2. J. Proteomics *118*, 49–62. https://doi.org/10.1016/j.jprot.2014.10.020.

26. Hong, H., and Benveniste, E.N. (2021). The Immune Regulatory Role of Protein Kinase CK2 and Its Implications for Treatment of Cancer. Biomedicines *9*, 1932. https://doi.org/10.3390/biomedicines9121932.

27. Roffey, S.E., and Litchfield, D.W. (2021). CK2 Regulation: Perspectives in 2021. Biomedicines *9*, 1361. https://doi.org/10.3390/biomedicines9101361.

28. Firnau, M.B., and Brieger, A. (2022). CK2 and the Hallmarks of Cancer. Biomedicines *10*, 1987. https://doi.org/10.3390/biomedicines10081987.

29. Herhaus, L., Perez-Oliva, A.B., Cozza, G., Gourlay, R., Weidlich, S., Campbell, D.G., Pinna, L.A., and Sapkota, G.P. (2015). Casein kinase 2 (CK2) phosphorylates the deubiquitylase OTUB1 at Ser16 to trigger its nuclear localization. Sci. Signal. *8*, ra35. https://doi.org/10.1126/scisignal.aaa0441.

30. Aregger, M., Kaskar, A., Varshney, D., Fernandez-Sanchez, M.E., Inesta-Vaquera, F.A., Weidlich, S., and Cowling, V.H. (2016). CDK1-Cyclin B1 Activates RNMT, Coordinating mRNA Cap Methylation with G1 Phase Transcription. Mol. Cell *61*, 734–746. https://doi.org/10.1016/j.molcel.2016.02.008.

31. Cozza, G., Mazzorana, M., Papinutto, E., Bain, J., Elliott, M., di Maira, G., Gianoncelli, A., Pagano, M.A., Sarno, S., Ruzzene, M., et al. (2009). Quinalizarin as a potent, selective and cell-permeable inhibitor of protein kinase CK2. Biochem. J. *421*, 387–395. https://doi.org/10.1042/BJ20090069.

32. Werner, M., Purta, E., Kaminska, K.H., Cymerman, I.A., Campbell, D.A., Mittra, B., Zamudio, J.R., Sturm, N.R., Jaworski, J., and Bujnicki, J.M. (2011). 2'-O-ribose methylation of cap2 in human: function and evolution in a horizontally mobile family. Nucleic Acids Res. *39*, 4756–4768. https://doi.org/10.1093/nar/gkr038.

33. Jumper, J., Evans, R., Pritzel, A., Green, T., Figurnov, M., Ronneberger, O., Tunyasuvunakool, K., Bates, R., Žídek, A., Potapenko, A., et al. (2021). Highly accurate protein structure prediction with AlphaFold. Nature *596*, 583–589. https://doi.org/10.1038/s41586-021-03819-2.

34. Varadi, M., Anyango, S., Deshpande, M., Nair, S., Natassia, C., Yordanova, G., Yuan, D., Stroe, O., Wood, G., Laydon, A., et al. (2022). AlphaFold Protein Structure Database: massively expanding the structural

coverage of protein-sequence space with high-accuracy models. Nucleic Acids Res. *50*, D439–D444. https://doi.org/10.1093/nar/gkab1061.

35. Galloway, A., Atrih, A., Grzela, R., Darzynkiewicz, E., Ferguson, M.A.J., and Cowling, V.H. (2020). CAP-MAP: cap analysis protocol with minimal analyte processing, a rapid and sensitive approach to analysing mRNA cap structures. Open Biol. *10*, 190306. https://doi.org/10.1098/rsob.190306.

36. Gitlin, L., Barchet, W., Gilfillan, S., Cella, M., Beutler, B., Flavell, R.A., Diamond, M.S., and Colonna, M. (2006). Essential role of mda-5 in type I IFN responses to polyriboinosinic:polyribocytidylic acid and encephalomyocarditis picornavirus. Proc. Natl. Acad. Sci. USA *103*, 8459–8464. https://doi.org/10.1073/pnas.0603082103.

37. Plotch, S.J., Bouloy, M., Ulmanen, I., and Krug, R.M. (1981). A unique cap(m7GpppXm)-dependent influenza virion endonuclease cleaves capped RNAs to generate the primers that initiate viral RNA transcription. Cell *23*, 847–858. https://doi.org/10.1016/0092-8674(81)90449-9.

38. Tsukamoto, Y., Hiono, T., Yamada, S., Matsuno, K., Faist, A., Claff, T., Hou, J., Namasivayam, V., Vom Hemdt, A., Sugimoto, S., et al. (2023). Inhibition of cellular RNA methyltransferase abrogates influenza virus capping and replication. Science *379*, 586–591. https://doi.org/10.1126/science.add0875.

39. Fukuyama, S., Katsura, H., Zhao, D., Ozawa, M., Ando, T., Shoemaker, J.E., Ishikawa, I., Yamada, S., Neumann, G., Watanabe, S., et al. (2015). Multi-spectral fluorescent reporter influenza viruses (Color-flu) as powerful tools for in vivo studies. Nat. Commun. *6*, 6600. https://doi.org/10.1038/ncomms7600.

40. Randall, R.E., and Goodbourn, S. (2008). Interferons and viruses: an interplay between induction, signalling, antiviral responses and virus countermeasures. J. Gen. Virol. *89*, 1–47. https://doi.org/10.1099/vir.0.83391-0.

41. Tran, V., Ledwith, M.P., Thamamongood, T., Higgins, C.A., Tripathi, S., Chang, M.W., Benner, C., García-Sastre, A., Schwemmle, M., Boon, A.C.M., et al. (2020). Influenza virus repurposes the antiviral protein IFIT2 to promote translation of viral mRNAs. Nat. Microbiol. *5*, 1490–1503. https://doi.org/10.1038/s41564-020-0778-x.

42. King, C.R., Liu, Y., Amato, K.A., Schaack, G.A., Mickelson, C., Sanders, A.E., Hu, T., Gupta, S., Langlois, R.A., Smith, J.A., and Mehle, A. (2023). Pathogen-driven CRISPR screens identify TREX1 as a regulator of DNA self-sensing during influenza virus infection. Cell Host Microbe *31*, 1552–1567.e8. https://doi.org/10.1016/j.chom.2023.08.001.

43. Furuichi, Y. (2015). Discovery of m(7)G-cap in eukaryotic mRNAs. Proc. Jpn. Acad. *91*, 394–409. https://doi.org/10.2183/pjab.91.394.

44. Meggio, F., and Pinna, L.A. (2003). One-thousand-and-one substrates of protein kinase CK2? FASEB J *17*, 349–368. https://doi.org/10.1096/fj.02-0473rev.

45. Borgo, C., and Ruzzene, M. (2021). Protein kinase CK2 inhibition as a pharmacological strategy. Adv. Protein Chem. Struct. Biol. *124*, 23–46. https://doi.org/10.1016/bs.apcsb.2020.09.003.

46. Salvi, M., Borgo, C., Pinna, L.A., and Ruzzene, M. (2021). Targeting CK2 in cancer: a valuable strategy or a waste of time? Cell Death Discov. *7*, 325. https://doi.org/10.1038/s41420-021-00717-4.

47. Borgo, C., D'Amore, C., Sarno, S., Salvi, M., and Ruzzene, M. (2021). Protein kinase CK2: a potential therapeutic target for diverse human diseases. Signal Transduct. Target. Ther. *6*, 183. https://doi.org/10.1038/s41392-021-00567-7.

48. Li, B., Clohisey, S.M., Chia, B.S., Wang, B., Cui, A., Eisenhaure, T., Schweitzer, L.D., Hoover, P., Parkinson, N.J., Nachshon, A., et al. (2020). Genome-wide CRISPR screen identifies host dependency factors for influenza A virus infection. Nat. Commun. *11*, 164. https://doi.org/10.1038/s41467-019-13965-x.

49. Bowie, A.G., and Unterholzner, L. (2008). Viral evasion and subversion of pattern-recognition receptor signalling. Nat. Rev. Immunol. *8*, 911–922. https://doi.org/10.1038/nri2436.

STAR★METHODS

KEY RESOURCES TABLE

REAGENT or RESOURCE	SOURCE	IDENTIFIER
Antibodies		
Anti-IFIT3 rabbit polyclonal	Proteintech	15201-1-AP; RRID:AB_2248738
Anti-ISG15 mouse monoclonal	Santa Cruz	sc-166755; RRID:AB_2126308
Anti-Actin mouse monoclonal	Santa Cruz	sc-47778; RRID:AB_626632
Anti-CMTR1 rabbit polyclonal	Sigma-Aldrich	HPA029954; RRID:AB_10670558
Anti-CMTR1 sheep polyclonal	University of Dundee	N/A
Anti-HA (Clone 16B12)	Biolegend	901514; RRID:AB_2565336
Anti-pCMTR1 sheep polyclonal	University of Dundee	N/A
Anti RNAPII 5SP rat polyclonal	Chromotek	3E8; RRID:AB_2631404
Anti RNA PII 2SP rat polyclonal	Chromotek	3E10; RRID:AB_2631403
Anti RNA PII (clone D8L4Y)	Cell Signaling	#14958; RRID:AB_2687876
Anti-DHX15 rabbit polyclonal	Abcam	ab254591; RRID:AB_2892059
Goat anti-Rabbit IgG (H + L) Secondary Antibody, HRP conjugate	Thermo Fisher	31460; RRID:AB_228341
Goat anti-Mouse IgG (H + L) Secondary Antibody, HRP conjugate	Thermo Fisher	31430; RRID:AB_228307
Rabbit anti-Sheep IgG (H + L) Secondary Antibody, HRP conjugate	Thermo Fisher	31480; RRID:AB_228457
Goat anti-Rat IgG (H + L) Secondary Antibody, HRP conjugate	Thermo Fisher	31470; RRID:AB_228356
IRDye® 680RD Donkey anti-Mouse IgG (H + L)	LI-COR Biotechnology	926–68072; RRID:AB_10953628
IRDye® 680RD Donkey anti-Rabbit IgG (H + L)	LI-COR Biotechnology	926–68073; RRID:AB_10954442
IRDye® 800CW Donkey anti-Rabbit IgG (H + L)	LI-COR Biotechnology	926–32213; RRID:AB_621848
IRDye® 680RD Donkey anti-Goat IgG (H + L)	LI-COR Biotechnology	926–68074; RRID:AB_10956736
Chemicals, Peptides, and Recombinant Proteins		
Recombinant human CMTR1 Full length	Division of Signal Transduction Therapies	N/A
Recombinant human CMTR1 Full length d1-143	Division of Signal Transduction Therapies	N/A
Recombinant CK2	Division of Signal Transduction Therapies	N/A
Recombinant OTUB1	Division of Signal Transduction Therapies	N/A
Deposited data		
RNA seq datasets	This paper	GSE124996 NCBI GEO database
Experimental models: Cell lines		
HeLa cells (human cervical cancer cell line)	ATCC	N/A
HEK 293 cells (Human embryonic kidney cell line)	ATCC	N/A
Experimental models: Primary Cell cultures		
Mouse Embryonic Fibroblasts (MEFs) *Cmtr1 fl/fl*	This paper	N/A
Experimental models: Organisms/Strains		
Cmtr1 fl/fl mice w/*loxP* sites flanking exon 3 of Cmtr1	Taconic Artemis Gmbh	N/A
Oligonucleotides		
IFIT1 F GAGGTTGTGCATCCCCAATG	This paper	N/A
IFIT1 R GCTACCACCTTTACAGCAACC	This paper	N/A

(*Continued on next page*)

Continued		
REAGENT or RESOURCE	SOURCE	IDENTIFIER
IFIT3 F TGGTTGCACACCCTGTCTTC	This paper	N/A
IFIT3 R GCAGCACAGAAACAGATCACC	This paper	N/A
IFIH1 F TTACACCTGACTCATTCCCGC	This paper	N/A
IFIH1 R CTGAGACTGCCCATGACGAG	This paper	N/A
ISG15 F AGTTAGTCACGGACACCAGGA	This paper	N/A
ISG15 R ACTCCTTAATTCCAGGGGACCTA	This paper	N/A
DHX58 F AGAGCTGTTGAGTGCCAACT	This paper	N/A
DHX58 R CAAGGTGGTGGTACTGGTCAA	This paper	N/A
Recombinant DNA		
pINI HA-CMTR1 WT	Division of Signal Transduction Therapies	N/A
pINI HA-CMTR1 15A (S26A, S28A, T30A, S31A, S46A, S49A, S51A, S53A, S55A, T57A, S63A, S64A, S66A, S75A, S89A)	Division of Signal Transduction Therapies	N/A
pINI HA-CMTR1 HA-CMTR1 T30A, S31A, S51A, S53A, S55A, T57A, S66A	Division of Signal Transduction Therapies	N/A
pINI HA-CMTR1 S26A, S28A, T30A, S31A, S51A, S53A, S55A, T57A, S64A, S66A, S75A, S89A	Division of Signal Transduction Therapies	N/A
pcDNA5FRTTO GFP CMTR1 WT	Division of Signal Transduction Therapies	N/A
pcDNA5FRTTO GFP CMTR1 K14E K15E K17E K18E (4K/E)	Division of Signal Transduction Therapies	N/A
pcDNA5FRTTO GFP CMTR1 25-835	Division of Signal Transduction Therapies	N/A
pCMV-FLAG CK2	Division of Signal Transduction Therapies	N/A
pCMV-FLAG CK2 D156A	Division of Signal Transduction Therapies	N/A
pBS598 EF1alpha-EGFPcre	Abcam	11923
Software and Algorithms		
TrimGalore (0.6.4)	N/A	https://github.com/FelixKrueger/TrimGalore/releases/tag/0.6.4
STAR (2.7.3a)	N/A	https://github.com/alexdobin/STAR/releases/tag/2.7.3a
Pic ard tool (2.23.3)	N/A	https://github.com/broadinstitute/picard/releases/tag/2.23.3
HTseq (0.12.4)	N/A	https://htseq.readthedocs.io/en/latest/install.html
edgeR (3.11)	N/A	https://bioconductor.riken.jp/packages/3.11/bioc/html/edgeR.html
Enrichr (3.0)	N/A	https://cran.r-project.org/src/contrib/Archive/enrichR/
ggplot2 (3.4.0)	N/A	https://cran.r-project.org/src/contrib/Archive/ggplot2/

RESOURCE AVAILABILITY

Lead contact

Information and requests for resources and reagents should be directed to and will be fulfilled by the lead contact, Victoria Cowling (victoria.cowling@glasgow.ac.uk).

Materials availability

Newly generated DNA constructs and other reagents are available on request from Victoria Cowling (victoria.cowling@glasgow.ac.uk).

Data and code availability

- RNA-seq data have been deposited at GEO and are publicly available as of the date of publication. Accession numbers are listed in the Key resources table.
- This paper does not report original code. Data processing as described in method detail.
- Any additional information required to reanalyze the data reported in this work paper is available from the lead contact upon request.

EXPERIMENTAL MODEL AND STUDY PARTICIPANT DETAILS

Animals

Cmtr1 fl/fl mice with *loxP* sites flanking exon 3 of Cmtr1 were sourced from Taconic Artemis Gmbh. Mice were maintained on a C57B6/J background in the Biological Resource Unit at the University of Dundee using procedures approved by the University Ethical Review Committee and under the authorization of the UK Home Office Animals (Scientific Procedures) Act 1986. Males and Females of age 8–20 weeks were used for breeding.

Cell lines

HeLa cells (human cervical cancer cell line).

HEK 293 cells (Human embryonic kidney cell line).

Cells were maintained subconfluent in DMEM (Dulbecco's modified Eagle's medium)/10%FCS (Fetal Calf Serum)/100U/ml penicillin, 0.1 mg/ml streptomycin and 2mM L-glutamine in 5% CO_2 at 37°C.

HeLa cells and HEK 293 cells made by retro-viral infection with pINI HA-CMTR1, HA-CMTR1 15A and pINI (protocol below). Infected cells were selected with 0.5 mg/ml G418 in the culture medium.

Primary cell cultures

MEF (Mouse Embryo Fibroblasts) were used.

To generate immortalised MEFs: *Cmtr1* $^{fl/fl}$ males and females were crossed. At day 13.5, embryos were removed from pregnant females, heads and internal organs removed and MEFs dissociated by trypsin digest. Insoluble material was removed by filtration. MEFs were cultured as above. MEFs were maintained subconfluent in DMEM (Dulbecco's modified Eagle's medium)/10%FCS (Fetal Calf Serum)/100U/ml penicillin, 0.1 mg/ml streptomycin and 2mM L-glutamine in 5% CO_2 at 37°C. MEFs were passage-immortalised.

METHOD DETAILS

Cell transfection and infection

Approximately 5×10^6 HeLa cells were transfected with 2μg pCDNA5 based constructs using Lipo2000 (Invitrogen). Cells were typically lysed after 1–3 days. For retroviral infection to create cell lines, Phoenix packaging cells were transfected with pINI vectors using Lipofectamine 2000. On day 1 medium was changed and on day 2 medium was taken off the cells and after filtration (0.22μM) applied onto the recipient cells with 5μg/ml polybrene (Sigma). On day 4 recipient cell medium was exchanged for the selection medium containing 0.5 mg/ml G418. When used, cells were incubated with 400U/ml interferon (Universal Type I IFN, PBL Assay Science) and 10μM Quinalizarin. Mouse embryonic fibroblasts were transfected with 5μg/ml poly(I:C) (Invivogen) using Lipofectamine 2000 (Invitrogen).

MEF immortalisation and Cre recombinase-based CMTR1 deletion

MEFs were transfected using GenJet *In Vitro* DNA Transfection Reagent for MEFs (SigneGen Laboratories, SL100489-MEF-05) with a vector expressing Cre-eGFP fusion protein under the control of the EF1α promoter. 24 h after transfection, the medium was changed to selection medium containing 2μg/mL puromycin and the cells were selected for 48 h, followed by 3–5 days of recovery in antibiotic-free medium. Deletion of endogenous *Cmtr1* was confirmed by PCR using the following primer pair: 5′-tgggtcccagacagtaaagg-3'/5′-cagtccagcaaagcatattcac-3′and Western Blotting.

Cell synchronisation

Nocodazole synchronisation and release: Subconfluent cells were cultured in 2.5mM thymidine for 24 h. Cells were washed with warmed culture medium 3 times prior to release into culture medium for 3hrs. 100ng/ul nocodazole was added for 13hrs. Cells were washed and released into culture. Thymidine synchronisation and release: Subconfluent cells were cultured in 2mM thymidine for 18 h, then washed 3 times in warmed medium prior to release into normal growth medium for 8 h. Cells were then cultured for a further 16hrs in 2mM thymidine, then washed 3 times prior to release into normal growth medium. Synchronisation and progression through the cell cycle was determined by staining cells with DAPI and FACS analysis of cell cycle progression. Cells were fixed in 4% PFA (Santa Cruz) for 30 min on ice, and permeabilised in 90% MeOH, 10% PBS for 30 min at −20°C. Cells were then washed in PBS, 2% FCS and resuspended in PBS, 2% FCS, 1 μg/mL DAPI. Cells were analyzed on a BD Fortessa flow cytometer and the data were analyzed using FlowJo v9.

Colorflu infection and FACS detection

MEF cell lines expressing CMTR1 WT, 15A or empty vector control (pINI) were infected at 80%–90% confluency with PR8 ColorFlu expressing a mCherry reporter gene fused to the viral NS1 gene at a MOI of 1 for 17 or 24hs. Post-infection, infected and mock-infected cells were collected into single cell suspension for analysis via flow cytometry using the LSRFortessa (BD Biosciences). The Percentage of infected cells was determined by calculating the percentage of mCherry positive cells within the total population of single alive cells. The average geometric mean of the mCherry signal in this population was also calculated. Analysis was carried out with FlowJo software version 10.9.0.

Mass spectrometry

HeLa cells were transfected with HA-CMTR1 WT or S/T to A mutants. 24hrs after transfection, cells were lysed in 50mM Tris/HCl pH 7.5, 0.5M NaCl, 1mM EGTA, 1mM EDTA, 1mM sodium orthovanadate, 10mM β-glycerophosphate, 50mM NaF, 5mM sodium pyrophosphate, 0.27M sucrose supplemented with 1% (v/v) Triton X-100, 2mM DTT, 1% (v/v) Apoprotin, 10μM Leupeptin and 1μM Pepstatin (all Sigma). Extracts were centrifuged at 16200 g at 4°C for 15 min and supernatant retained. Cell extracts were subject to immunoprecipitation using anti-HA agarose affinity gel (Sigma). IPs were resolved by SDS-PAGE and proteins stained with Novex Colloidal blue (Invitrogen). Bands corresponding to the size of CMTR1 were excised and washed sequentially on vibrating platform with 0.5mL water, 1:1(v/v) water and acetonitrile (AcN), 0.1M ammonium bicarbonate, 1:1(v/v) 0.1M ammonium bicarbonate and AcN. Protein samples were reduced in 10mM dithiothreitol (20 min, 37°C), alkylated in 50mM iodoacetamide/0.1M ammonium bicarbonate (20 min, dark), and washed in 50mM ammonium bicarbonate and 50mM ammonium bicarbonate/50% AcN. On becoming colourless, gel pieces were washed with AcN for 15min and dried. Gel pieces were digested by incubation in 5 μg/ml trypsin/25mM triethylammonium bicarbonate (16h, 30°C). Tryptic digests were analyzed by liquid chromatography–mass spectrometry on Applied Biosystems 4000 QTRAP system (Foster City, CA) with precursor ion scanning. Resulting MS/MS data searched using the Mascot search algorithm (http://www.matrixscience.com).

In vitro kinase assay

200ng CK2a, 1μg substrate (CMTR1 or OTUB1) and 10 μM ^{32}P ATP (50cpm/pmole), was incubated at 30°C for 30 min or the time indicated, in 50mM Tris/HCl, pH 7.5, 0.1 mM EGTA, 10mM $MgCl_2$, 0.1% beta mercaptoethanol. The reaction was stopped by dilution in Lamelli buffer. Products were resolved on 10% SDS PAGE, stained with Coommassie Blue and exposed to film. When indicated, IPs were incubated with 20U/μl Lambda phosphatase for 1 h at 30°C.

Molecular biology

In vitro mutagenesis was performed using the Qiagen Quick Change mutagenesis protocol by the Division of Signal Transduction Therapies. Constructs were sequence verified by Sanger Sequencing.

Cell lysis and Co-immunoprecipitation

Cells were lysed in lysis buffer (10mM TRIS pH 7.05, 50mM NaCl, 30mM Na4 pyrophosphate, 50mM NaF, 5μM ZnCl2, 10% glycerol, 0.5% Triton X-100 1mM DTT; supplemented with protease and phosphatase inhibitors) and cleared by centrifugation. For immunoprecipitation (IP) of HA-tagged proteins, anti-HA antibody-conjugated agarose (Sigma) or Pierce Anti-HA Magnetic Beads were used. 10–20 μL antibody beads were incubated with 0.1-1mg protein for 1-2 hrs at 4°C. Beads were washed 3–5 times in lysis buffer, then resuspended in 30-50uls 2x loading buffer, and 8-15ul was resolved by SDS-PAGE. Endogenous CMTR1 immunoprecipitation was conducted as above except, 1μg anti-CMTR1 antibody (sheep polyclonal) was pre-incubated with 10μL protein-G Sepharose packed beads and washed to remove non-bound antibody. 0.5–2.5mg cell extract was precleared with 10-30μL protein-G Sepharose (GE Healthcare), then incubated with antibody–bound beads for 2 h at 4°C, prior to bead washing and protein elution.

Antibody production

pCMTR1 antibodies were raised in sheep by the Division of Signal Transduction Therapies, University of Dundee. The human CMTR1 peptide, 23LALSL**S**S**TS**DDEPP36, phosphorylated on S28, T30 and S31 was the immunogen. Sera were purified on this peptide.

Dot blot

Recombinant CMTR1 was dropped onto PVDF and allowed to dry. Blots were probed using a western blotting protocol. Briefly blots were probed with polyclonal anti-pCMTR1 antibody or (pan) CMTR1 antibody (in-house), washed, then probed with secondary antibody: HRP-linked anti-sheep antibodies and washed again. ECL (Pierce) was used to visualise HRP-antibody binding.

Western Blot and quantification

Western blotting was performed by standard protocols using antibodies indicated and bands quantitated using ImageJ software.

RNA sequencing analysis

MEF RNA was extracted using GenJET RNA purification kit (ThermoFisher). The quality and quantity of RNA were assessed using Tapestation (Agilent) and Qubit (ThermoFisher) measurements. The libraries were prepared using rRNA depletion protocol and

sequenced by Novogene using mRNA Seq Illumina PE150, Q30 ⩾ 80% (30M reads, 9Gb data). Lexogene SIVR-Set3 spike-ins were used.

The reads were quality and adapter trimmed using TrimGalore (0.6.4) before the read alignment. Then the processed reads were aligned to the mouse reference genome GRCm38/mm10 using STAR (2.7.3a). For the aligned reads, PCR duplicates were removed using Picard tool (2.23.3). Then the reads on each transcript were quantified using HTseq (0.12.4) package. Libraries normalization and differential expression analysis were performed using the exactTest in edgeR (3.11). Only transcripts expressed above the threshold of 3 reads per million in all replicates were considered for analysis. The gene ontology of the differentially expressed genes were analyzed using Enrichr (3.0). The dot plots and boxplots of RNA-seq data were generated using ggplot2 (3.4.0). All analysis packages were run using the default settings from their user manuals.

QUANTIFICATION AND STATISTICAL ANALYSIS

Statistical Analysis was performed as described in the figure legends using processing tools in GraphPad Prism 10 or Microsoft Excel. *p* values are stated on the figures. Where relevant, the number of independent experiments performed is indicated. Independent experiments with respect to tissue culture mean cells treated and harvested on independent occasions.

Cell Reports, Volume *43*

Supplemental information

CK2 phosphorylation of CMTR1 promotes RNA cap formation and influenza virus infection

Radoslaw Lukoszek, Francisco Inesta-Vaquera, Natasha J.M. Brett, Shang Liang, Lydia A. Hepburn, David J. Hughes, Chiara Pirillo, Edward W. Roberts, and Victoria H. Cowling

A

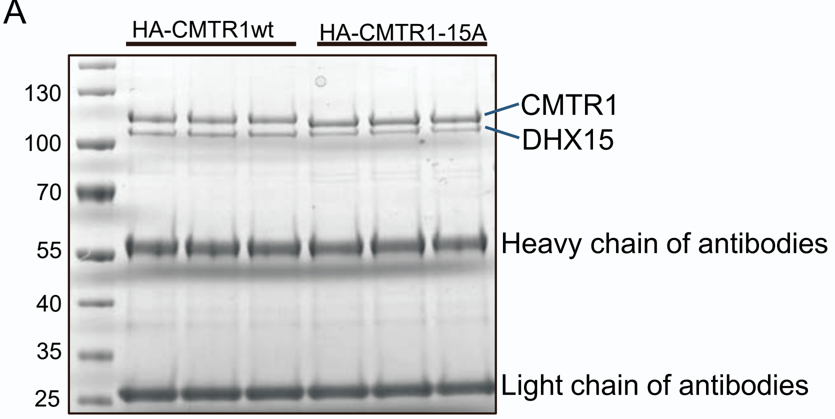

B

HA-CMTR1 WT peptide coverage (90% coverage)

1	MYPYDVPDYA	MKRRTDPECT	APIKKQKKRV	AELALSLSST	SDDEPPSSVS
51	HGAKASTTSL	SGSDSETEGK	QHSSDSFDDA	FKADSLVEGT	SSRYSMYNSV
101	SQKLMAKMGF	REGEGLGKYS	QGRKDIVEAS	SQKGRRGLGL	TLRGFDQELN
151	VDWRDEPEPS	ACEQVSWFPE	CTTEIPDTQE	MSDWMVVGKR	KMIIEDETEF
201	CGEELLHSVL	QCKSVFDVLD	GEEMRRARTR	ANPYEMIRGV	FFLNRAAMKM
251	ANMDFVFDRM	FTNPRDSYGK	PLVKDREAEL	LYFADVCAGP	GGFSEYVLWR
301	KKWHAKGFGM	TLKGPNDFKL	EDFYSASSEL	FEPYYGEGGI	DGDGDITRPE
351	NISAFRNFVL	DNTDRKGVHF	LMADGGFSVE	GQENLQEILS	KQLLLCQFLM
401	ALSIVRTGGH	FICKTFDLFT	PFSVGLVYLL	YCCFERVCLF	KPITSRPANS
451	ERYVVCKGLK	VGIDDVRDYL	FAVNIKLNQL	RNTDSDVNLV	VPLEVIKGDH
501	EFTDYMIRSN	ESHCSLQIKA	LAKIHAFVQD	TTLSEPRQAE	IRKECLRLWG
551	IPDQARVAPS	SSDPKSKFFE	LIQGTEIDIF	SYKPTLLTSK	TLEKIRPVFD
601	YRCMVSGSEQ	KFLIGLGKSQ	IYTWDGRQSD	RWIKLDLKTE	LPRDTLLSVE
651	IVHELKGEGK	AQRKISAIHI	LDVLVLNGTD	VREQHFNQRI	QLAEKFVKAV
701	SKPSRPDMNP	IRVKEVYRLE	EMEKIFVRLE	MKIIKGSSGT	PKLSYTGRDD
751	RHFVPMGLYI	VRTVNEPWTM	GFSKSFKKKF	FYNKKTKDST	FDLPADSIAP
801	FHICYYGRLF	WEWGDGIRVH	DSQKPQDQDK	LSKEDVLSFI	QMHRA

HA-CMTR1 15A peptide coverage (91% coverage)

1	MYPYDVPDYA	MKRRTDPECT	APIKKQKKRV	AELALALASA	ADDEPPSSVS
51	HGAKAATTAL	AGADAEAEGK	QHAADAFDDA	FKADALVEGT	SSRYSMYNAV
101	SQKLMAKMGF	REGEGLGKYS	QGRKDIVEAS	SQKGRRGLGL	TLRGFDQELN
151	VDWRDEPEPS	ACEQVSWFPE	CTTEIPDTQE	MSDWMVVGKR	KMIIEDETEF
201	CGEELLHSVL	QCKSVFDVLD	GEEMRRARTR	ANPYEMIRGV	FFLNRAAMKM
251	ANMDFVFDRM	FTNPRDSYGK	PLVKDREAEL	LYFADVCAGP	GGFSEYVLWR
301	KKWHAKGFGM	TLKGPNDFKL	EDFYSASSEL	FEPYYGEGGI	DGDGDITRPE
351	NISAFRNFVL	DNTDRKGVHF	LMADGGFSVE	GQENLQEILS	KQLLLCQFLM
401	ALSIVRTGGH	FICKTFDLFT	PFSVGLVYLL	YCCFERVCLF	KPITSRPANS
451	ERYVVCKGLK	VGIDDVRDYL	FAVNIKLNQL	RNTDSDVNLV	VPLEVIKGDH
501	EFTDYMIRSN	ESHCSLQIKA	LAKIHAFVQD	TTLSEPRQAE	IRKECLRLWG
551	IPDQARVAPS	SSDPKSKFFE	LIQGTEIDIF	SYKPTLLTSK	TLEKIRPVFD
601	YRCMVSGSEQ	KFLIGLGKSQ	IYTWDGRQSD	RWIKLDLKTE	LPRDTLLSVE
651	IVHELKGEGK	AQRKISAIHI	LDVLVLNGTD	VREQHFNQRI	QLAEKFVKAV
701	SKPSRPDMNP	IRVKEVYRLE	EMEKIFVRLE	MKIIKGSSGT	PKLSYTGRDD
751	RHFVPMGLYI	VRTVNEPWTM	GFSKSFKKKF	FYNKKTKDST	FDLPADSIAP
801	FHICYYGRLF	WEWGDGIRVH	DSQKPQDQDK	LSKEDVLSFI	QMHRA

Figure S1, related to Figure 1. Peptide coverage in CMTR1 mass spectrometry analysis

Human HA-CMTR1 WT and 15A (mutant with 15 alanine substitutions, see text) were expressed in HeLa cells, immunoprecipitated via the HA tag and analysed by mass spectrometry. A) SDS PAGE analysis. Protein visualized by Coomassie stain. CMTR1 and binding partner DHX15 indicated. For each (IP), 3 replicates were performed. B) The peptides identified are indicated in red on the human CMTR1 amino acid sequence.

HA-CMTR1 T30A, S31A, S51A, S53A, S55A, T57A, S66A				
**Peptides Identified by mass spectrometry**	**MH+ [Da]**	**Mascot score**	**Possible sites**	**% Probability**
YSMYNSVSQK	1286.50957	48	S89	98.9
ASTTSLAGADAEAEGK	1558.65837	53	S51	84.2
VAELALSLSSAADDEPPSSVSHGAK	2518.16196	85	S26	99.9
QHSSDAFDDAFKADSLVEGTSSR	2550.06882	28	S75	100

HA-CMTR1 S26A, S28A, T30A, S31A, S51A, S53A, S55A, T57A, S64A, S66A, S75A, S89A				
**Peptides Identified by mass spectrometry**	**MH+ [Da]**	**Mascot score**	**Possible sites**	**% Probability**
ASTTSLAGADAEAEGK	1558.66814	49	S49	99.3
ASTTSLAGADAEAEGKQHSADAFDDAFK	2891.25034	34	S63	100

HA-CMTR1 S26A, S28A, T30A, S31A, S46A, S49A, S51A, S53A, S55A, T57A, S63A, S64A, S66A, S75A, S89A (15A)				
**Peptides Identified by mass spectrometry**	**MH+ [Da]**	**Mascot score**	**Possible sites**	**% Probability**
No phosphorylated peptides detected.				

Figure S2, related to Figure 1. CMTR1 phospho-site mapping

The HA-CMTR1 alanine mutants indicated were transiently expressed in HeLa cells and immunoprecipitated via the HA tag. Phopho-peptides were identified by mass spectrometry, mass reported. The most likely phosphorylation sites are stated in “possible sites” with % probability given.

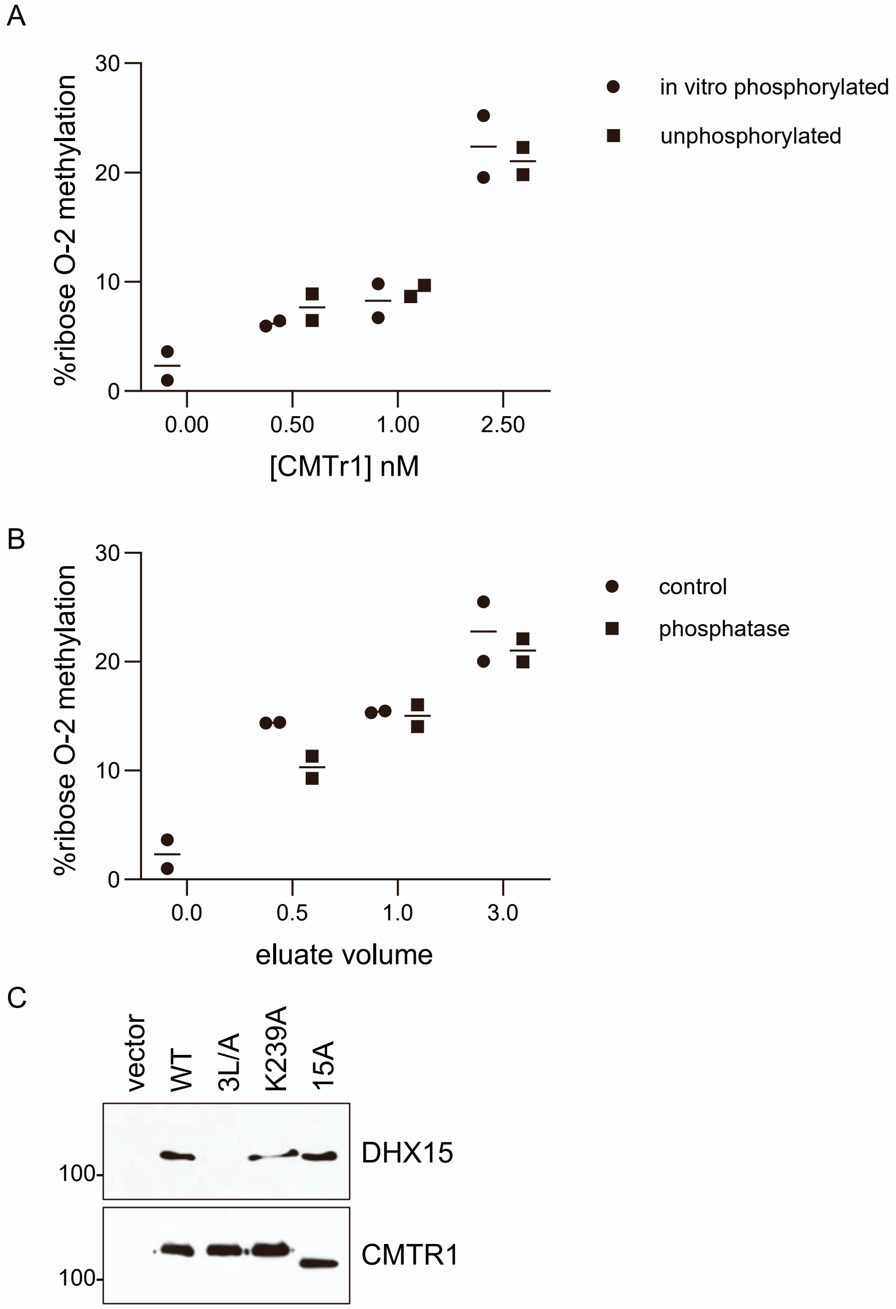

Figure S3, related to Figure 2. CMTR1 phosphorylation does not significantly alter methyltransferase activity or DHX15 interaction

A) Recombinant CMTR1 was phosphorylated *in vitro* by CK2 or mock control (see methods). B) HA-CMTR1 was immunoprecipitated from HeLa cells, eluted and subject to Lambda phosphatase treatment or mock control. For A and B) Ribose O-2 methylation was quantified by *in vitro* assay. Bar represents the average. C) HA-CMTR1 WT, 3L/A, K239A (catalytic dead mutant) and 15A were transiently expressed in HeLa cells. HA- CMTR1 complexes were immunoprecipitated and analysed by western blot for CMTR1 and DHX15 components.

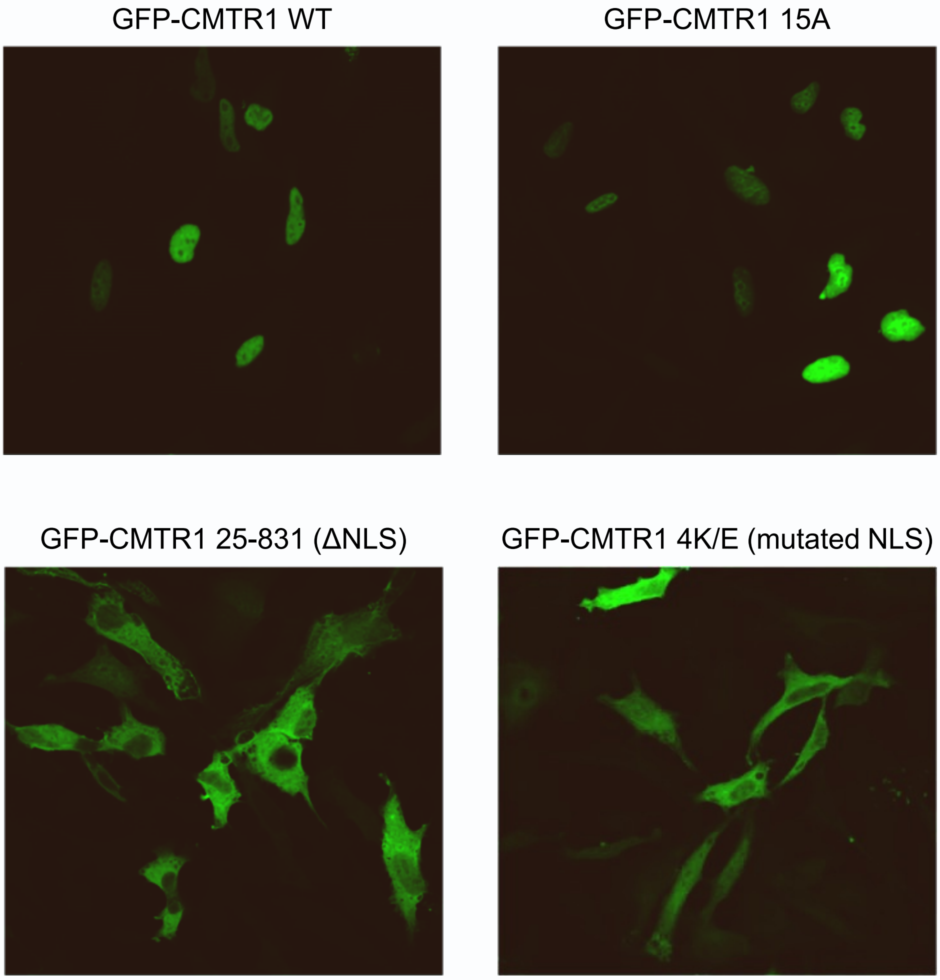

Figure S4, related to Figure 2. GFP-CMTR1 WT and 15A have a diffuse nuclear localisation. GFP-CMTR1 WT, 15A, 25-831 (ΔNLS, nuclear localisation sequence) and 4K/E (mutated NLS) were expressed in HeLa cells and visualised by fluorescence microscopy.

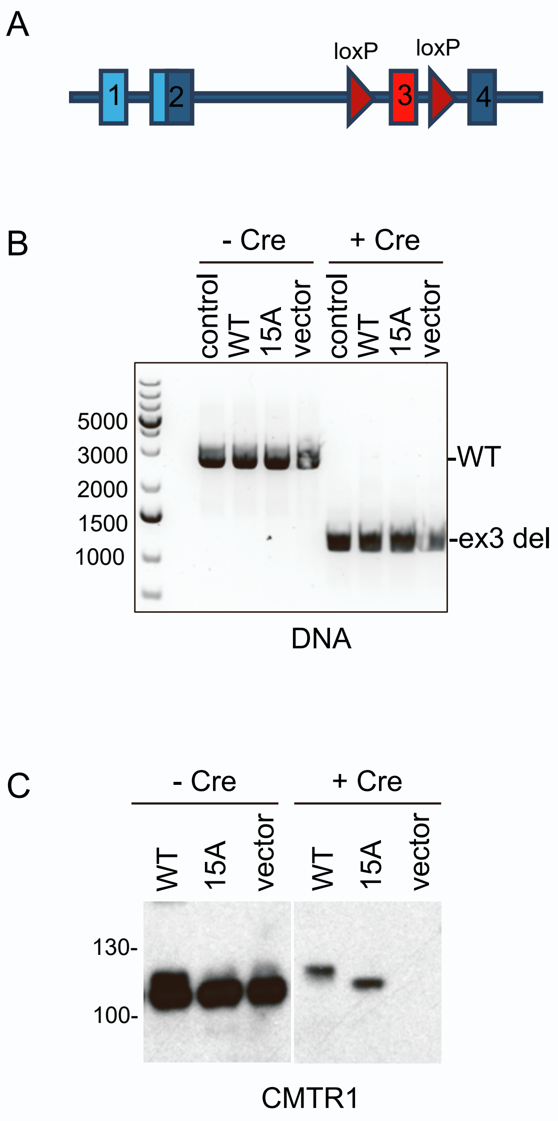

Figure S5, related to Figure 5. Cmtr1 floxed gene

a) Diagram of floxed *Cmtr1* gene in mouse embryonic fibroblasts (MEFs). Exons numbered and loxP sites indicated. Exon 3 (red) is deleted by expression of Cre recombinase, resulting in a frameshift. MEF lines were created to express CMTR1 WT, 15A and vector control. Cre recombinase was expressed transiently. b) PCR of genomic DNA surrounding exon 3. WT product and exon 3 deleted product after expression of Cre recombinases indicated. c) Cell extracts were analysed for CMTR1 expression by western blot before (-Cre) and after (+Cre) Cre mediated deletion. The CMTR1 antibody recognises endogenous CMTR1 and HA-CMTR1.

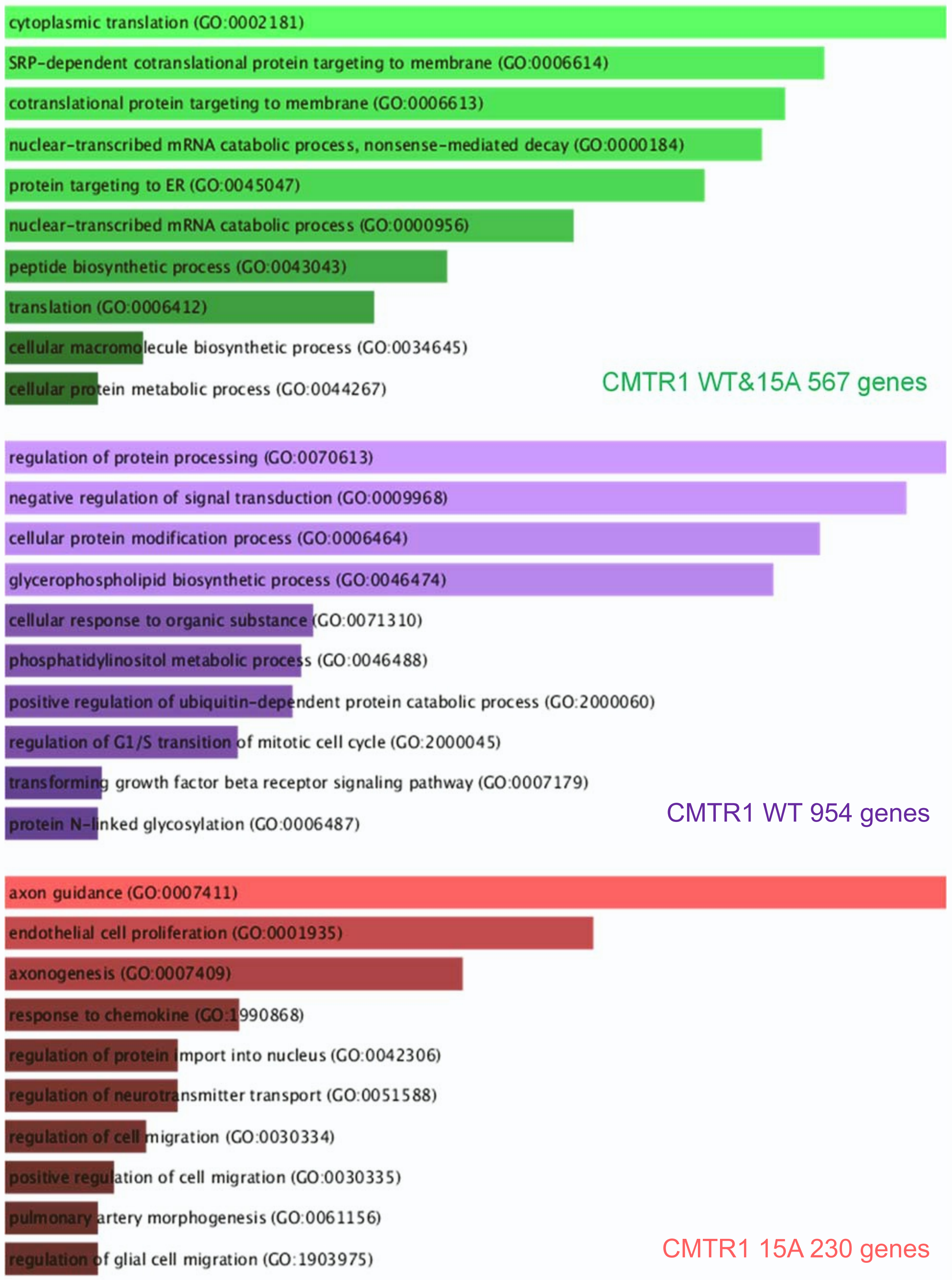

Figure S6, related to Figure 5. GO Term analysis of CMTR1 regulated genes

Genes regulated in response to CMTR1 WT, CMTR1 15A or both in Cmtr1 -/- MEFs. The number of genes in each data set indicated.
